# The rs599839 A>G Variant Disentangles Cardiovascular Risk and Hepatocellular Carcinoma in NAFLD Patients

**DOI:** 10.3390/cancers13081783

**Published:** 2021-04-08

**Authors:** Marica Meroni, Miriam Longo, Erika Paolini, Anna Alisi, Luca Miele, Emilia Rita De Caro, Giuseppina Pisano, Marco Maggioni, Giorgio Soardo, Luca Vittorio Valenti, Anna Ludovica Fracanzani, Paola Dongiovanni

**Affiliations:** 1 General Medicine and Metabolic Diseases, Fondazione IRCCS Ca’ Granda Ospedale Maggiore Policlinico, 20122 Milano, Italy; marica.meroni@unimi.it (M.M.); miriam.longo@unimi.it (M.L.); erika.paolini@unimi.it (E.P.); emilia.decaro@policlinico.mi.it (E.R.D.C.); Giuseppina.pisano@policlinico.mi.it (G.P.); anna.fracanzani@unimi.it (A.L.F.); 2 Department of Pathophysiology and Transplantation, Università degli Studi di Milano, 20122 Milano, Italy; luca.valenti@unimi.it; 3 Department of Clinical Sciences and Community Health, Università degli Studi di Milano, 20122 Milano, Italy; 4 Department of Pharmacological and Biomolecular Sciences, Università degli Studi di Milano, 20133 Milano, Italy; 5 Research Unit of Molecular Genetics of Complex Phenotypes, Bambino Gesù Children Hospital, IRCCS, 00165 Rome, Italy; anna.alisi@opbg.net; 6 Area Medicina Interna, Gastroenterologia e Oncologia Medica, Fondazione Policlinico Universitario A. Gemelli IRCCS, 00168 Rome, Italy; luca.miele@policlinicogemelli.it; 7 Department of Pathology, Fondazione IRCCS Ca’ Granda Ospedale Maggiore Policlinico, 20122 Milano, Italy; marco.maggioni@policlinico.mi.it; 8 Clinic of Internal Medicine-Liver Unit Department of Medical Area (DAME), University School of Medicine, Udine, Italy and Italian Liver Foundation AREA Science Park—Basovizza Campus, 34149 Trieste, Italy; giorgio.soardo@asuiud.sanita.fvg.it; 9 Precision Medicine, Department of Transfusion Medicine and Hematology, Fondazione IRCCS Ca’ Granda Ospedale Maggiore Policlinico, 20122 Milano, Italy

**Keywords:** lipid metabolism, NAFLD, genetic variants, PSRC1, HCC

## Abstract

**Simple Summary:**

Dyslipidemia is a hallmark of nonalcoholic fatty liver disease (NAFLD) and the rs599839 variant in the *CELSR2-PSRC1-SORT1* genetic cluster, has been associated with a protection against cardiovascular events. Here, we revealed a novel link between the rs599839 variant and hepatocellular carcinoma (HCC) whose onset in the context of NAFLD is rapidly increasing. We found that the rs599839 variant disentangled the risk of HCC from that of cardiovascular abnormalities by modulating SORT1 and PSRC1 expressions. The latter emerged as a potential modifier of liver carcinogenesis.

**Abstract:**

Background and Aims: Dyslipidemia and cardiovascular diseases (CVD) are comorbidities of nonalcoholic fatty liver disease (NAFLD), which ranges from steatosis to hepatocellular carcinoma (HCC). The rs599839 A>G variant, in the CELSR2-PSRC1-SORT1 gene cluster, has been associated CVD, but its impact on metabolic traits and on the severity liver damage in NAFLD has not been investigated yet. Methods: We evaluated the effect of the rs599839 variant in 1426 NAFLD patients (Overall cohort) of whom 131 had HCC (NAFLD-HCC), in 500,000 individuals from the UK Biobank Cohort (UKBBC), and in 366 HCC samples from The Cancer Genome Atlas (TCGA). Hepatic PSRC1, SORT1 and CELSR2 expressions were evaluated by RNAseq (*n* = 125). Results: The rs599839 variant was associated with reduced circulating LDL, carotid intima-media thickness, carotid plaques and hypertension (*p* < 0.05) in NAFLD patients and with protection against dyslipidemia in UKBBC. The minor G allele was associated with higher risk of HCC, independently of fibrosis severity (odds ratio (OR): 5.62; 95% c.i. 1.77–17.84, *p* = 0.003), poor prognosis and advanced tumor stage (*p* < 0.05) in the overall cohort. Hepatic PSRC1, SORT1 and CELSR2 expressions were increased in NAFLD patients carrying the rs599839 variant (*p* < 0.0001). SORT1 mRNA levels negatively correlated with circulating lipids and with those of genes involved in lipoprotein turnover (*p* < 0.0001). Conversely, PSRC1 expression was positively related to that of genes implicated in cell proliferation (*p* < 0.0001). In TCGA, PSRC1 over-expression promoted more aggressive HCC development (*p* < 0.05). Conclusions: In sum, the rs599839 A>G variant is associated with protection against dyslipidemia and CVD in NAFLD patients, but as one it might promote HCC development by modulating SORT1 and PSRC1 expressions which impact on lipid metabolism and cell proliferation, respectively.

## 1. Introduction

Nonalcoholic fatty liver disease (NAFLD) or as more recently redefined, metabolic-dysfunction associated fatty liver disease (MAFLD), is the most common chronic liver disorder worldwide, affecting an estimated 20–40% of the adult population [1,2]. Thus, given its increasing prevalence, it represents a primary health, social and economic concern [3]. NAFLD is defined by excessive hepatic fat accumulation not explained by alcohol abuse, and it embraces a broad spectrum of hepatic injuries, spanning from simple steatosis to nonalcoholic steatohepatitis (NASH). The latter may be complicated by the development of fibrosis and, in a minor percentage of cases, it may progress to cirrhosis and hepatocellular carcinoma (HCC), that is the fifth most common cancer worldwide with persistently increasing mortality in Europe, North/South America and Africa [4]. NAFLD and its progressive forms have a strong inherited component, and variants in genes regulating lipid handling, including patatin-like phospholipase domain-containing 3 (*PNPLA3*), transmembrane 6 superfamily member 2 (*TM6SF2*) and membrane bound O-acyltransferase domain-containing 7 (*MBOAT7*), predispose to NAFLD development and progression towards end-stage conditions [5,6]. Moreover, sedentary lifestyle and unhealthy dietary habits may represent paramount environmental risk factors for NAFLD pathogenesis in predisposed individuals [7]. Hence, its development is closely intertwined with obesity, insulin resistance (IR) and metabolic syndrome features among which atherogenic dyslipidemia and cardiovascular risk [8,9].

However, despite the epidemiological association between NAFLD and cardiovascular disease, we previously shown that genetic variants impacting on lipoprotein secretion and determining hepatocellular lipid retention, such as loss-of-function variants in *TM6SF2*, predispose to progressive NAFLD despite offering protection against cardiovascular disease [10]. Therefore, we hypothesized that other variants with a large impact on lipoprotein metabolism may help to stratify the relative risk of cardiovascular vs. liver-related events in patients with NAFLD, which is urgently awaited in order to refine clinical management strategies for this condition.

A genome wide association study (GWAS) identified the novel intergenic rs599839 A>G variant, as modifier of the risk of coronary artery disease (CAD) [11]. Afterwards, the more frequent A allele has been associated with increased risk of myocardial infarction, ischemic stroke and elevated plasma cholesterol, thereby modulating lipoprotein metabolism [12,13]. Conversely, the minor G allele has been related to a protection against cardiovascular complications and to a reduction of circulating cholesterol in CAD patients [14].

The rs599839 polymorphism is localized in the 1p13.3 locus related to lipid traits, in the genetic cluster of cadherin EGF LAG seven-pass G-type receptor 2 (*CELSR2*)—proline and serine rich coiled-coil 1 (*PSRC1*)—sortilin 1 (*SORT1*). In details, the *CELSR2* gene encodes a non-classic type of cadherin involved in cell adhesion [15], while the *PSRC1* gene product participates to microtubule destabilization and spindle assembly [16] and its overexpression has been detected in tumor liver tissues and in hepatoma cells, where it is associated with HCC recurrence after resection [17]. Finally, the *SORT1* gene encodes the sortilin 1 protein that is involved in lipoprotein clearance [18]. The impact of the rs599839 variant on CELSR2, PSRC1 and SORT1 expressions remain to be fully elucidated. However, these genes are involved in distinct pathways, mainly related to lipid handling, and dysregulated cell proliferation, and alterations in their expressions may possibly affect different processes.

Since atherogenic dyslipidemia is a hallmark of NAFLD and the rs599839 variant has been associated with reduced circulating lipids in patients with CAD, we firstly aimed to examine the effect of the rs599839 variant on metabolic traits, cardiovascular risk and progressive liver damage in a large histologically characterized cohort of NAFLD patients at risk of cardiovascular comorbidities. Secondly, we evaluated the impact of the rs599839 mutation on dyslipidemia and CAD risk in the general population by using the publicly available UK biobank cohort (UKBBC). Then, we assessed whether the rs599839 variant modulates the hepatic expression of *PSRC1*, *SORT1* and *CELSR2* and of those genes involved in lipoprotein turnover and release, in cell cycle progression and cell proliferation. Finally, we investigated whether the overexpression of the oncogene PSRC1 contributes to a more severe HCC phenotype in The Cancer Genome Atlas (TCGA) dataset.

## 2. Materials and Methods

### 2.1. Overall Cohort

The overall cohort consisted of 1426 patients with NAFLD, and it was subdivided into the hepatology service cohort (*n* = 1295) and the NAFLD-HCC cohort (*n* = 131). The hepatology service cohort has been described previously [10,19]. Briefly, 1295 adult individuals were consecutively enrolled at the metabolic liver diseases outpatient service (*n* = 713) and the bariatric surgery center (*n* = 582) at Fondazione IRCCS Ca’ Granda Ospedale Policlinico (Milan, Italy). Inclusion criteria were availability of liver biopsy performed for suspected NASH or severe obesity, availability of DNA samples and clinical data. Individuals with excessive alcohol intake (men, >30 g/day; women, >20 g/day), viral and autoimmune hepatitis, hereditary haemochromatosis and alpha1-antitrypsin deficiency or other causes of liver disease were excluded. The clinical characteristics of patients evaluated in the study are listed in Table 1.

The NAFLD-HCC cohort includes 131 NAFLD patients who developed HCC. Part of this cohort has been previously described [20,21]. Patients were consecutively enrolled at the metabolic liver diseases outpatient service at Fondazione IRCCS Cà Granda, Ospedale Policlinico, at the internal medicine and gastroenterology area, Fondazione Policlinico Universitario Gemelli and IRCCS, Catholic University (Rome, Italy) and at the internal medicine-liver unit, University Hospital of Udine (Udine, Italy). Clinical features of the NAFLD-HCC cohort are presented in Table 1. Diagnosis of HCC was based on the EASL–EORTC Clinical Practice Guidelines [22]. In the absence of liver biopsy, diagnosis of NAFLD required detection of ultrasonographic steatosis plus at least one criterion of the metabolic syndrome.

Informed written consent was obtained from each patient and the study protocol was approved by the Ethical Committees of the Fondazione IRCCS Ca’ Granda, Milan, of Fondazione Policlinico Universitario Gemelli and IRCCS, Catholic University, Rome, and from University Hospital of Udine and conforms to the ethical guidelines of the 1975 Declaration of Helsinki.

### 2.2. Histological Evaluation

Steatosis was graded according to the percentage of affected hepatocytes as 0: 0–4%, 1: 5–32%, 2: 33–65%, and 3: 66–100%. Disease activity was assessed according to the NAFLD Activity Score (NAS) with systematic evaluation of hepatocellular ballooning and necroinflammation; fibrosis was also staged according to the recommendations of the NAFLD Clinical Research Network [23]. The scoring of liver biopsies was performed by independent pathologists unaware of patients’ status and genotype [10,24]. NASH was diagnosed when (a) steatosis, (b) lobular inflammation and (c) ballooning were concomitantly present.

### 2.3. Genotyping

The overall cohort has been genotyped for the rs738409 C>G (PNPLA3 I148M), rs58542926 C>T (TM6SF2 E167K), rs641738 C>T *MBOAT7*, and rs599839 A>G variants using TaqMan 5′-nuclease assays in duplicate (QuantStudio 3, Thermo Fisher, Waltham, MA, USA), as previously described [10,19]. The success rate of genotyping was >99%. The frequency distribution of the rs599839 A>G was not in Hardy-Weinberg equilibrium (*p* = 0.01, Table S1) and it was compared to that obtained in European not-Finnish healthy individuals included in the 1000 Genome project [25].

### 2.4. UK Biobank Cohort

The association between the rs599839 A>G *PSRC1* variant and phenotypes related to metabolic disorders and liver disease were evaluated in the UK Biobank cohort (UKBBC). UKBBC is a prospective population-based study of approximately 500,000 individuals not selected for liver diseases and ethnicity, almost all aged 40–69 years, identified in 22 centers across the UK during 2006–2010. Freely available basic association data were downloaded from Neale Lab in March 2020 (http://geneatlas.roslin.ed.ac.uk) and *p*-Values were corrected for multiple testing using the false discovery rate (FDR) method [26].

### 2.5. Transcriptomic Analysis

RNA-seq was performed in a subset of 125 severely obese patients (21 without and 104 with NAFLD) belonging to the hepatology service cohort, of whom percutaneous liver biopsy was performed during bariatric surgery at Fondazione IRCCS Cà Granda, Ospedale Policlinico [27]. The study was conformed to the Declaration of Helsinki and approved by the Institutional Review Boards and their Ethics Committees. All participants gave written informed consent. Clinical characteristics of the transcriptomic cohort are presented in Table S2. RNA-seq mapping descriptive statistics, the detailed protocol and data analysis approach are described in the Supplemental Materials and Methods.

### 2.6. The Cancer Genome Atlas-Liver Hepatocellular Carcinoma (TCGA-LIHC) Data Description

The Cancer Genome Atlas-Liver Hepatocellular Carcinoma (TCGA-LIHC) database is a large project which applies high-throughput genome analysis techniques, combining genome sequencing and bioinformatic tools, in order to catalogue genetic mutations responsible for cancer. It is a comprehensive publicly available resource, that contains information about expression level of multiple genes. TCGA datasets of 366 HCC samples were directly downloaded from cBioPortal for Cancer Genomics. The detailed information of the microarray and RNA-Seq experiments, protocols, and software used can be found at the cBioPortal for Cancer Genomics at https://www.cbioportal.org [28,29]. Data were downloaded in March 2020. Relative mRNA expression was represented as Z scores, precomputed from the expression values specifying the threshold (two standard deviations from the mean). The z scores for mRNA expression are determined for each sample by comparing a gene’s mRNA expression to the distribution in a reference population that represents typical expression of that gene. TCGA tumor stage system classification and bioinformatic resources are described in the Supplemental Materials and Methods.

### 2.7. Statistical Analysis

Statistical analyses were performed using JMP 15.0 (SAS, Cary, NC), R statistical analysis version 3.3.2 (http://www.R-project.org/) and Prism (version 6, GraphPad Software Inc, San Diego, CA, USA), by using one-way analysis of variance (ANOVA) or chi-square test, where appropriate.

For descriptive statistics, continuous variables were shown as mean and standard deviation or median and interquartile range for highly skewed biological variables (i.e., AST, ALT, triglycerides (TGs)). Variables with skewed distributions were logarithmically transformed before analyses. Categorical variables were presented as number and proportion. All genetic analyses were performed under additive and recessive models.

Analyses were performed by fitting data to generalized linear regression models. Generalized linear models were fit to examine continuous traits. Multinomial logistic regression models were fit to examine binary traits (cirrhosis, HCC), and ordinal regression models were fit for ordinal traits (components of the NAFLD activity score: severity of steatosis, necroinflammation and hepatocellular ballooning, stage of fibrosis). When specified, confounding factors were included in a model. Correlations were assessed by bivariate analysis. For gene expression analyses differences between groups were calculated by one-way ANOVA, which was followed by post hoc *t*-tests adjusted for the number of comparisons when multiple groups were involved (Bonferroni correction or Benjamini-Hochberg false discovery rate (FDR) correction, where indicated). *p* values <0.05 (two-tailed) were considered statistically significant.

## 3. Results

### 3.1. The rs599839 A>G Gene Variant Affects Circulating Lipids and Cardiovascular Risk in NAFLD Patients

**Figure 1:**
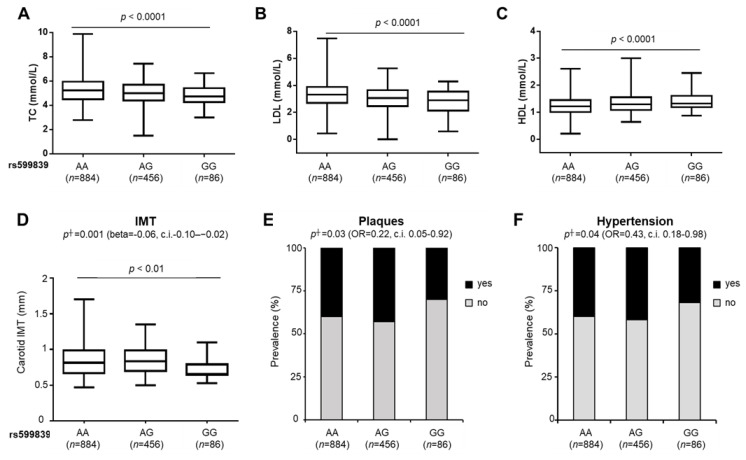
The rs599839 variant affects circulating lipids, carotid IMT, plaque formation and incidence of hypertension in patients with NAFLD. Circulating total cholesterol (TC) (mmol/L) (**A**), LDL (mmol/L) (**B**), HDL (mmol/L) (**C**) were evaluated in NAFLD patients from the Overall cohort (*n* = 1426) and stratified by the presence of the rs599839 G allele. Boxes span from 25° to 75° percentile, while whiskers indicate the 10° and 90° percentile. *p* < 0.0001 at one-way ANOVA. Association of the rs599839 variant with IMT (**D**), plaque presence (**E**) and hypertension (**F**) in NAFLD patients from the Overall cohort (*n* = 1426). Multivariable generalized linear model (for IMT) or nominal logistic regression analysis (for Plaques and Hypertension) adjusted for age, sex, BMI, T2D, presence of TM6SF2 E167K, statin use and active smoking at † recessive model. *p* < 0.01 at one-way ANOVA.

Clinical characteristics of the overall cohort stratified according to the rs599839 A>G variant are shown in Table 2. No differences in demographic and anthropometric features were found across genotypes. Circulating total cholesterol (TC) and low-density lipoprotein (LDL) cholesterol were reduced in NAFLD patients across the rs599839 genotype, while high-density lipoprotein (HDL) cholesterol levels were higher in patients who carry the minor G allele (*p* < 0.001 at one-way ANOVA; adjusted *p* < 0.0001 for GG vs. AA, Figure 1A–C). To sum up these results, the severity of dyslipidemia was reduced in patients harboring the rs599839 minor allele (*p =* 0.02 at Pearson test, Figure S1A).

At multivariate generalized linear models, we found that the G minor allele was associated with reduced TC (beta: −0.15; 95% c.i. −0.25–−0.04; *p* = 0.005), LDL cholesterol (beta: −0.20; 95% c.i. −0.30–−0.10; *p* < 0.0001) and with increased HDL cholesterol (beta: 0.07; 95% c.i. 0.03–0.10; *p* = 0.0003), after adjustment for sex, age, body mass index (BMI), type 2 diabetes (T2D), PNPLA3 I148M, TM6SF2 E167K and *MBOAT7* rs641738 variants by using an additive model (Table 2). Consistently, at multivariate analysis, the prevalence of dyslipidemia was lower in patients who carry the minor G allele (OR: 0.76; 95% c.i. 0.60–0.95; *p* = 0.01), adjusted for the same confounders (Table 2 and Figure S1A).

At recessive model, LDL cholesterol (beta: −0.14; 95% c.i. −0.27–−0.009; *p* = 0.03), along with dyslipidemia (OR: 0.37; 95% c.i. 0.18–0.77; *p* = 0.007) remained associated with the rs599839 variant (Table 2 and Figure S1A). Conversely, we did not find any association between the rs599839 variant and circulating TGs, T2D or IR.

Therefore, we next evaluated the impact of the rs599839 G allele on cardiovascular comorbidities in the Overall cohort. At multivariate generalized linear models, it was associated with reduced carotid intima-media thickness (IMT) (beta: −0.06; 95% c.i. −0.10-−0.02; *p* = 0.001), carotid artery plaque formation (OR: 0.22; 95% c.i. 0.05–0.92; *p* = 0.03) and lower prevalence of hypertension (beta: 0.43; 95% c.i. 0.18–0.98; *p* = 0.04) after adjustment for sex, age, BMI, T2D, TM6SF2 E167K variant, statin use and active smoking only under a recessive model (Figure 1D–F and Table 3).

### 3.2. The rs599839 A>G Variant Is Not Associated with Histological NAFLD

Since the rs599839 variant influences circulating lipid concentrations, which are strongly entangled in hepatic fat accumulation, we analyzed its impact on liver damage in NAFLD patients from the overall cohort. At ordinal regression analysis, the rs599839 variant was not significantly related to steatosis, lobular inflammation, ballooning, fibrosis (Figure S1A–D) and cirrhosis neither at additive or recessive models (Table 4 and Tables S3 and S4), thus suggesting that the G allele did not impact on in the histological spectrum of NAFLD. This observation differs from what we observed for the E167K TM6SF2 variant, which by impairing VLDL release, induces fat accumulation in the liver, fibrosis and at the same time protects against CAD [10].

### 3.3. The rs599839 Variation Is Associated with Increased Risk of HCC in NAFLD Patients

Since the rs599839 variant is located at 500 b downstream of the 3′ untranslated region (UTR) of *PSRC1* gene, which is required for the congress of chromosomes at the metaphase plate and for normal rate of chromosomal segregation during anaphase, we next sought to examine whether the rs599839 variation may affect the risk to develop HCC in NAFLD patients. Patients affected by HCC in the context of NAFLD are characterized by a higher frequency of biological sex male (78% vs. 52%; *p* < 0.0001 at one-way ANOVA), are older (mean age 68 vs. 48 years; *p* < 0.0001 at one-way ANOVA) and display an increased incidence of T2D (57% vs. 24%; *p* < 0.0001 at one-way ANOVA) compared to those subjects without cancer. As consequence of advanced liver disease, these patients are characterized by severely enhanced circulating ALT and AST levels (*p* = 0.07 and *p* < 0.0001 at one-way ANOVA, respectively). In addition, according to literature evidence [30,31], HCC patients display an increased frequency distribution of *PNPLA3* variant (29% MM vs. 16%; *p* < 0.0001 at one-way ANOVA) compared to NAFLD patients without cancer.

**Figure 2:**
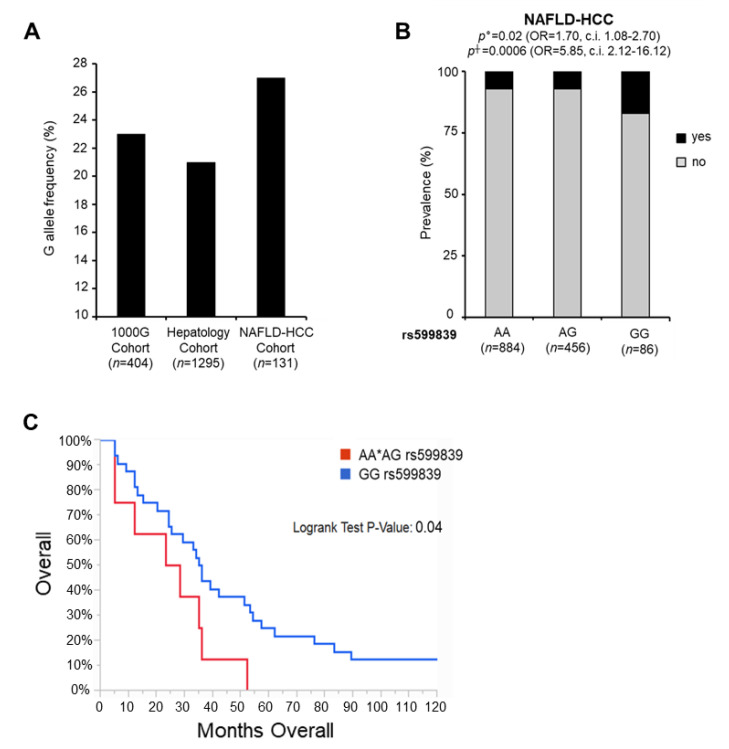
The rs599839 variant influences the risk of HCC in NAFLD patients. Frequencies distribution of the rs599839 variant across the 404 individuals from 1000 genomes European non-Finnish cohort, Hepatology service cohort (*n* = 1295) and NAFLD-HCC cohort (*n* = 131) (**A**). Association of the rs599839 variant with HCC (*n* = 131 cases from the NAFLD-HCC cohort and *n* = 1295 controls with NAFLD from the Hepatology service cohort). Multivariable nominal logistic regression analysis adjusted for age, sex, BMI, T2D, presence of PNPLA3 I148M, TM6SF2 E167K and MBOAT7 T alleles at ° additive or † recessive model (**B**). Kaplan-Meier survival analysis of patients carrying the rs599839 in homozygosity (*n* = 16; GG rs599839—red line) vs. the others (*n* = 115; AA*AG rs599839—blue line). X axis indicates the months of survival. Log-rank Test *p*-Value: 0.04 (**C**).

The frequency distribution of the minor G allele in the Hepatology service cohort (*n* = 1295) and in the NAFLD-HCC cohort (*n* = 131) is shown in Figure 2A, in Table 1 and in Table S1. The minor G allele was more frequent in NAFLD patients with HCC than in those without cancer (*p* = 0.01 at one-way ANOVA (Table 1); 27% vs. 21% *p* = 0.03 at Fisher-exact Test NAFLD-HCC vs. Hepatology service cohort; Figure 2A and Table S1) and the percentage of GG homozygous patients was even higher in NAFLD-HCC cohort (12% vs. 5% NAFLD-HCC vs. Hepatology service cohort; Table 1). Furthermore, at multivariate analysis, the G allele was independently associated with enhanced risk of HCC at both additive (OR: 1.70; 95% c.i. 1.08–2.70; *p* = 0.02) and recessive models (OR: 5.85; 95% c.i. 2.12–16.12; *p* = 0.0006). Notably, the effect of the variant on HCC development was independent of the liver disease severity, as adjustment for advanced fibrosis did not abolish the association between the G allele and HCC at both models (OR: 1.70; 95% c.i. 1.03–2.80; *p* = 0.03; and OR: 5.62; 95% c.i. 1.77–17.84; *p* = 0.003, respectively) (Figure 2B and Table 5). Furthermore, at multivariate analysis, the rs599839 variant was associated with an increased risk to develop tumors diagnosed at an advanced stage (Stage (S) >1; OR: 3.27; 95% c.i. 1.36–7.85; *p* = 0.008) and more pronounced primary tumor extensions (Tumor size (T) >1; OR: 2.86; 95% c.i. 1.17–6.96; *p* = 0.02). Consistently, the at-risk G allele correlated with higher Child-Pugh scores (Child-Pugh >A6; OR: 4.38; 95% c.i. 1.35–14.22; *p* = 0.01) (Table 6). Since the presence of rs599839 GG homozygosis is most strongly associated with aggressive HCC, we deeply investigated the differences in the overall survival and in the number of deceases, between homozygous HCC patients and the others. We revealed that in patients carrying the GG the average months of survival was 24.50, while in the others 45.60, although the mean age of diagnosis is 67 years for both groups. Moreover, at 10-years follow up (120 months) the number of deceases in patients carrying the GG was 14 (88%), whereas in the others 71 (62%) (Log-rank Test *p* = 0.04) (Figure 2C). Collectively, these findings suggest that the rs599839 variant might increase the risk to develop aggressive HCC in NAFLD patients independently of hepatic fat accumulation or fibrosis, and more so in homozygous subjects.

### 3.4. The rs599839 A>G Gene Variant Affects Circulating Cholesterol and CAD Risk but Not Liver Damage in UKBBC

The protective effect of the rs599839 G allele on lipid metabolism and cardiovascular risk was confirmed by analyzing the data obtained from the UKBBC. Indeed, there was a strong negative association between the rs599839 G allele and circulating TC levels (beta: −0.017; *p* = 7.82 × 10^−112^), disorders of lipoprotein metabolism (beta: −0.01; *p* = 4.3 × 10^−55^), metabolic disorders (beta: −0.01; *p* = 3.09 × 10^−44^) and the presence of cardiovascular complications such as ischemic heart diseases (beta: −0.006; *p* = 6.72 × 10^−25^), hypertensive diseases (beta: −0.002; *p* = 0.018) and atherosclerosis (beta: −0.0003; *p* = 0.037). The clinical phenotypes related to rs599839 variant in the UKBBC are listed in Table 7. Remarkably, the rs599839 variant was not correlated with liver failure and hepatic fibrosis and cirrhosis thus confirming what we found in NAFLD patients.

### 3.5. Association between rs599839 and Other Genetic Variants at the 1p13.3 Locus

To investigate whether the associations between the rs599839 polymorphism and metabolic traits were due to other common variants (MAF ≥ 0.01) located in the 1p13.3 *locus*, we examined the linkage disequilibrium pattern at the region spanning 50.00 Kb (chr1: 109,800,000–109,850,000; Human (GRCh37.p13)), using data from 1000 genomes project and considering 503 individuals of European descent (CEU). The rs599839 variant resulted in strong linkage disequilibrium with other 10 common SNPs (rs1277930, rs583104, rs4970836, rs602633, rs7528419, rs629301, rs646776, rs12740374, rs3832016, rs660240) (r^2^ > 0.8; Table S5), localized at the region that ranges from the *CELSR2* 3′UTR, the intergenic region between *CELSR2* and *PSRC1*, and the *PSRC1* 3′UTR oriented in opposite direction (Figure S2A). Thus, we evaluated the clinical phenotypes most significantly associated with these SNPs in the UKKBC and we found that all these variants were negatively correlated with circulating TC concentrations (beta: −0.017; *p* < 0.0001), supporting previous data from Musunuru et al. [12] on the effect of the entire 1p13.3 *locus* on circulating lipids and confirming the protective role of the rs599839 minor G allele on lipid metabolism even in NAFLD patients.

### 3.6. The rs599839 A>G Impacts on PSRC1, SORT1 and CELSR2 Expression

**Figure 3:**
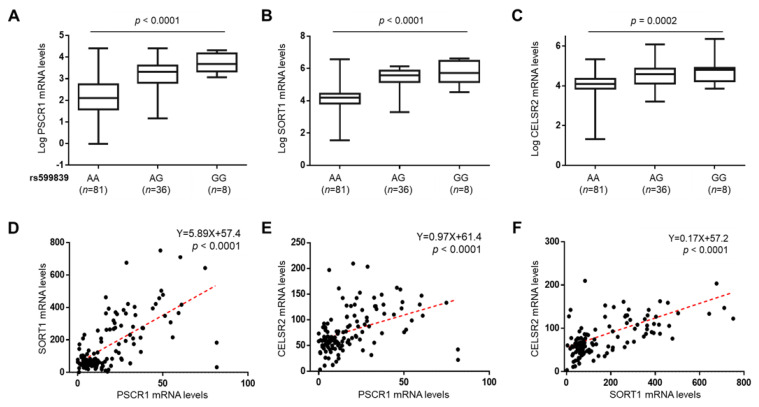
PSRC1, SORT1 and CELSR2 expressions increase in NAFLD patients carrying the rs599839 variant. PSRC1 (**A**), SORT1 (**B**) and CELSR2 (**C**) mRNA levels were evaluated by transcriptome analysis on liver biopsies (*n* = 125) and stratified by the presence of the rs599839 G allele. mRNA levels were represented as log transformed. Boxes span from 25° to 75° percentile, while whiskers indicate the 10° and 90° percentile. *p* < 0.001 at one-way ANOVA. Correlation analyses between PSRC1 and SORT1 (**D**), CELSR2 (**E**) and between SORT1 and CELSR2 (**F**).

To determine whether the epidemiological association between the rs599839 variant and protection against CAD and higher risk to develop HCC in NAFLD patients may be mediated by the modulation of transcriptional activity at this locus, we examined the hepatic expression of PSRC1, SORT1 and CELSR2 in a subset of patients belonging to the Overall cohort of whom transcriptomic data was available (*n* = 125). The rs599839 G allele was associated with higher PSRC1 (*p* < 0.0001 at one-way ANOVA; adjusted *p* < 0.0001 for GG vs. AA), SORT1 (*p* < 0.0001 at one-way ANOVA; adjusted *p* < 0.0001 for GG vs. AA) and CELSR2 (*p =* 0.0002 at one-way ANOVA; adjusted *p* < 0.001 for GG vs. AA) mRNA levels (Figure 3A–C). In addition, there was a strong positive correlation between their expressions (*p* < 0.0001; for all comparisons) (Figure 3D–F). As we expected, PSRC1, SORT1 and CELSR2, which are predicted to interact according to a network analysis generated by STRING, were significantly co-expressed (*p* = 8.44 × 10^−7^, Figure S2B), indicating that these proteins are at least partially biologically connected, as a group.

**Figure 4:**
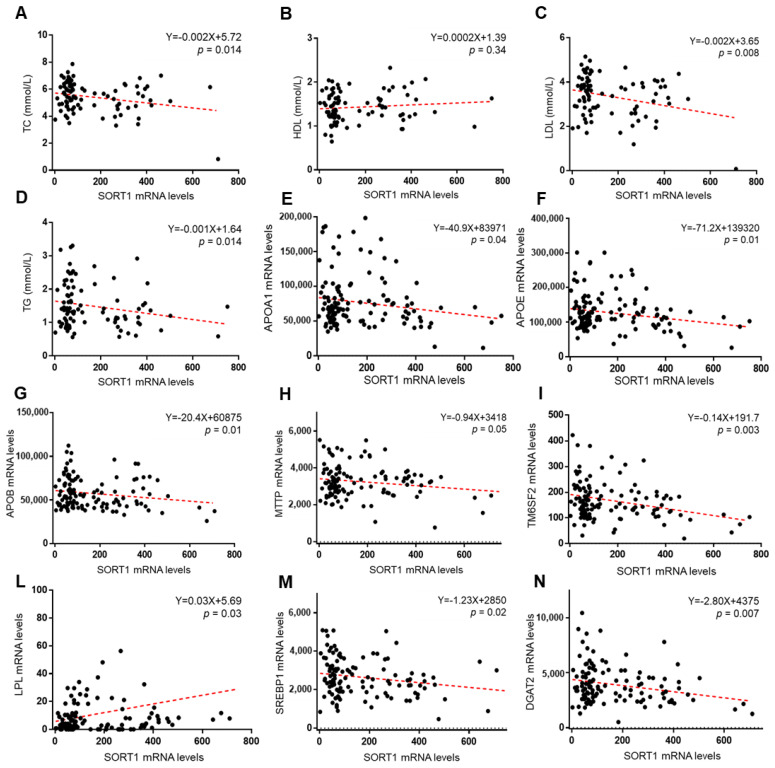
SORT1 mRNA levels correlate with the concentration of circulating lipids and with the expression of genes involved in lipoprotein release and lipid synthesis. Correlation analyses between hepatic SORT1 gene expression evaluated by transcriptome analysis on liver biopsies (*n* = 125) and circulating total cholesterol (TC) (mmol/L) (**A**), LDL (mmol/L) (**B**), HDL (mmol/L) (**C**), and triglycerides (mmol/L) (**D**). Correlation analyses between hepatic SORT1 gene expression and APOA1 (**E**), APOE (**F**), APOB (**G**), MTTP (**H**), TM6SF2 (**I**), LPL (**L**), SREBP1 (**M**), DGAT2 (**N**) mRNA levels evaluated by transcriptome analysis on liver biopsies (*n* = 125).

Our data is consistent with previous findings which indicate that SORT1 is the primary mediator of circulating lipids at this locus. Indeed, SORT1 expression, but not that of PSRC1 or CELSR2, was significantly correlated with the reduction of TC concentrations (Y = −0.002X + 5.72; *p* = 0.014), and more strongly with the decrease in LDL cholesterol (Y = −0.002X + 3.65; *p* = 0.0008) and serum TGs (Y = −0.001X + 1.64; *p* = 0.014). However, SORT1 mRNA levels did not correlate with HDL cholesterol (Figure 4A–D). Even more, the expression of SORT1 was tightly correlated with that of apolipoprotein A1 (APOA1; Y = −40.9X + 83971; *p* = 0.04), apolipoprotein E (APOE; Y = −71.2X + 139320; *p* = 0.01), APOB (Y = −20.4X + 60875; *p* = 0.01), microsomal triglyceride transfer protein (MTTP; Y = −0.94X + 3418; *p* = 0.05), TM6SF2 (Y = −0.14X + 191.7; *p* = 0.003), Lipoprotein lipase (LPL; Y = 0.03X + 5.69; *p* = 0.03), sterol regulatory element-binding protein−1 (SREBP1; Y = −1.23X + 2850; *p* = 0.02) and diacylglycerol O-acyltransferase 2 (DGAT2; Y = −2.80X + 4375; *p* = 0.007) genes, thus, reinforcing the role of SORT1 in lipoprotein turnover, lipid synthesis and dismissal (Figure 4E–N). Conversely, PSRC1 mRNA levels more marginally impacted on the expression of these genes (Figure S3A,B), while CELSR2 expression did not impact at all. This data suggests that the alteration of SORT1 expression, induced by presence of the rs599839 variant, is the main driver of the reduced lipid concentrations observed in patients who carry the variant.

**Figure 5:**
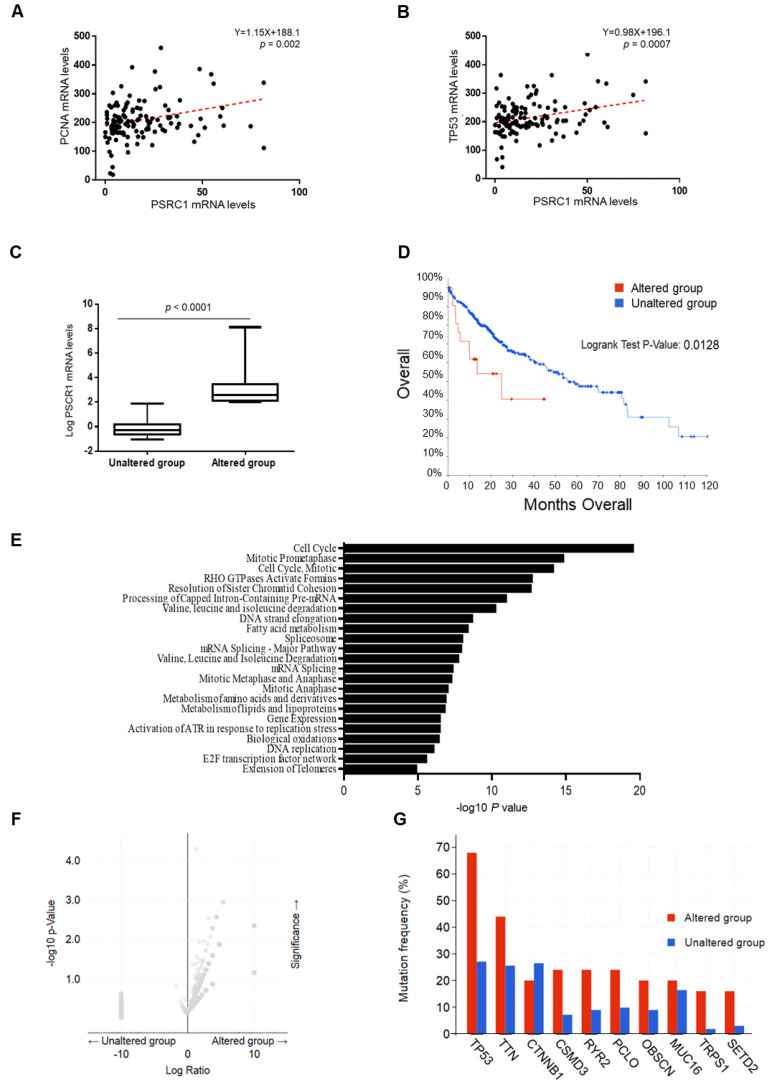
PSRC1 mRNA levels correlate with that of genes involved in cell proliferation and its overexpression reduced survival in HCC patients. Correlation analyses between hepatic PSRC1 gene expression and PCNA (**A**) and TP53 (**B**) mRNA levels evaluated by transcriptome analysis on liver biopsies (*n* = 125). PSRC1 mRNA levels were evaluated by transcriptome analysis on HCC samples from TCGA dataset (*n* = 366). Among the 366 HCC samples, 25 patients (7%) overexpressed hepatic PSRC1 (altered group) compared to the others (*n* = 341, unaltered group). mRNA levels were represented as log transformed. Boxes span from 25° to 75° percentile, while whiskers indicate the 10° and 90° percentile. *p* < 0.0001 at two-tailed Student *t*-test (**C**). Kaplan-Meier survival analysis of patients with PSRC1 overexpressed (*n* = 25; altered group—red line) vs. the others (*n* = 341; unaltered—blue line). X axis indicates the months of survival. Log-rank Test *p* value: 0.0128 (**D**). Reactome pathways enriched for 6353 genes co-regulated with PSRC1 expression in 366 samples from TCGA dataset. The statistical significance level (*p*-Value) was negative 10-based log trans-formed (**E**). Volcano plot illustrates the differential frequency distribution of mutations, observed in patients belonging to the altered group (*n* = 25) compared to the unaltered one (*n* = 341), represented as log fold changes (log ratio, on x axis) and the distribution of transformed *p* values (−log10 of *p*, y axis) (**F**). The frequency of mutations in the 10 genes most enriched in mutations in both groups are stratified according to the presence of PSRC1 overexpression (*n* = 25, altered group in red) or not (*n* = 341, unaltered group in blue) (**G**).

To further explore the association between the rs599839 variant and HCC risk, we investigated the relationship between the expression of the *PSRC1-SORT1-CELSR2* gene cluster and that of genes involved in on cell proliferation. We found that PSRC1 mRNA levels positively correlated with those of proliferating cell nuclear antigen (PCNA; Y = 1.15X + 188.1; *p* = 0.002) and tumor protein p53 (TP53; Y = 0.98X + 196.1; *p* = 0.0007) (Figure 5A,B). Conversely, SORT1 expression was less strongly associated with that of PCNA and TP53 (Figure S3C,D), whereas the one of CELSR2 did not correlate with the expression of genes involved in cell cycle progression. In sum, the association of the rs599839 variant with HCC risk in NAFLD patients seems to be most likely accounted by enhanced PSCR1 expression.

### 3.7. PSRC1 Overexpression Aggravates HCC Features and Prognosis in TCGA

To further endorse the role of PSRC1 in hepatic carcinogenesis we assessed its hepatic expression in 366 HCC patients enrolled in TCGA dataset. Among them, 25 (7%) displayed an overexpression of hepatic PSRC1 (altered group) compared to the others (*n* = 341, unaltered group) (*p* < 0.0001 at *t*-tests; Figure 5C). Likewise, independently of ethnicity, gender and age at diagnosis, PSRC1, but not SORT1 or CELSR2 mRNA levels, were independently associated with more advanced tumor stage (S >1; beta: 0.7 ± 0.06; 95% C.I. 0.05–0.27; *p* = 0.006), severe histological grade of liver cancer (G > 2; beta: 0.17 ± 0.06; 95% C.I. 0.05–0.29; *p* = 0.005) and primary tumor extension (T > 1; beta: 0.17 ± 0.05; c.i. 0.06–0.28; *p* = 0.003) (Figure S4A–C and Table 8). These findings suggest that tumors overexpressing PSRC1 tend to be more undifferentiated, grow rapidly and spread faster to nearby and distal tissues, further corroborating the results obtained in NAFLD-HCC patients who carried the G minor allele. Consequently, in patients in whom PSRC1 was overexpressed, the average of months of survival was 24.89, while in the others was 55.69. Likewise, the total number of deceases in the altered group was 10 (40%), whereas in the unaltered one it was 119 (35%) (Log-rank Test *p =* 0.0128) (Figure 5D).

Consistently with the results obtained from the transcriptomic cohort, PSRC1 mRNA levels positively correlated with the expression of SORT1 and CELSR2 and with well-established proliferation markers such as PCNA, MKI67, TERT, CDC20, numerous cyclins (CCNs), cyclin dependent kinases (CDK1–4), genes involved in DNA replication (MCM2–10, E2F2) and many other oncogenes (Table S6). However, we did not observe any correlation between TP53 expression and PSRC1 in TCGA. Conversely, PSRC1 expression negatively correlated with that of genes involved in lipoprotein release and cholesterol synthesis (such as APOB and DGAT2) (Table S6). In details, a pathway-enriched analysis of the 6353 genes most significantly co-regulated with PSRC1, confirmed that these genes are mainly involved in cell cycle progression (*q* = 1.07 × 10^−19^ at Benjamini-Hochberg FDR correction) (Figure 5E and Table S7).

We next analyzed the frequency rate of pathogenic gene mutations in patients who belong to the altered group compared to the unaltered one, as shown in the volcano plot in Figure 5F. Among the most mutated genes, we highlighted that *TP53* had the highest rate of mutation in the group of patients with altered expression of PSRC1. Indeed, among patients belonging to the altered group, 17 cases (68%) displayed *TP53* putative driver mutations, including 10 missense and seven truncating pathogenic mutations, while among the others (unaltered group) only 91 (27%) showed mutation in *TP53* (*p* = 5.11 × 10^−5^). Along with *TP53* several other genes implicated in cell proliferation (*CTNNB1* (*p* = 0.32), *CSMD3 (p* = 0.01), *PCLO* (*p* = 0.04), *MUC16* (*p* = 0.40), *TRPS1* (*p* = 2.8 × 10^−3^) and *SETD2* (*p* = 0.01)) or cardiovascular damage (*TTN* (*p* = 0.04), *RYR2* (*p* = 0.028), *OBSCN* (*p* = 0.08)) have been found to be enriched in mutation rate in the altered group (Figure 5G).

Finally, to investigate which genes were deregulated in the context of PSRC1 overexpression, we performed a gene expression differential analysis of the whole hepatic transcriptome of patients belonging to the altered group compared to the unaltered one. We found 3794 differentially expressed genes, 2129 being up-regulated and 1665 down-regulated in patients bearing HCC with high PSRC1 expression (*q* < 0.05, at Benjamini-Hochberg FDR correction). Pathway-enriched analysis confirmed that up-regulated genes were mainly involved in cell proliferation (Figure S5A,B and Table S8), whereas down-regulated in metabolic pathways, metabolism of lipids and lipoproteins and in mitochondrial fatty acid β-oxidation (Figure S6A,B and Table S9). Overall, these findings may support the hypothesis that the hyper-activation of PSRC1 transcription is linked and may promote tumor growth, de-differentiation and invasion and they may explain the loss of significant correlation between PSRC1 and TP53 in patients carrying PSRC1 altered expression, as an escape from the mechanism of tumor suppression.

## 4. Discussion

In this study, we examined the impact of the rs599839 A > G variant which is localized in the genetic cluster of *CELSR2*-*PSRC1*-*SORT1* on metabolic phenotypes and liver damage in a large cohort of histologically characterized NAFLD patients, in individuals from UKKBC and in HCC patients from TCGA. We found that the minor G allele was associated with protection against atherogenic dyslipidemia, carotid plaque formation and hypertension in NAFLD patients, which are at higher risk of cardiovascular comorbidities. Consistently, the presence of the G allele was related to protection against hypercholesterolemia and several cardiovascular outcomes also in the population based UKKBC.

The rs599839 variant has been previously identified through GWAS and then validated in epidemiological studies as a genetic factor protecting against CAD, myocardial infarction, abdominal aortic aneurysm and lipid traits [11,32,33,34,35,36]. Its impact on CAD risk factors has been further validated in the Sikh Diabetes Study [37,38] and in the Indian Atherosclerosis Research Study (IARS) [14]. Furthermore, the association between GG homozygosity and the protection against arterial hypertension has been confirmed in a cohort of 5460 Japanese individuals [39].

The rs599839 variant is in strong linkage disequilibrium with other 10 common SNPs at the 1p13.3 locus (rs1277930, rs583104, rs4970836, rs602633, rs7528419, rs629301, rs646776, rs12740374, rs3832016, rs660240), all localized in the genomic region ranging from the 3′UTR of *CELR2*, the intergenic region and the *PSRC1* 3′UTR oriented in opposite direction. As expected, given the strong linkage disequilibrium, all these SNPs have been found to be correlated with reduced TC levels, with the same direction, effect size and strength of the rs599839 variant. Thus, the impact of the entire 1p13.3 locus on TC modulation which has been observed in previous studies may be translated even in NAFLD patients.

Despite its large impact on circulating cholesterol levels, the rs599839 variant did not have a significant impact on hepatic fat accumulation, nor on lobular inflammation, ballooning or fibrosis. The lack of any significant association between liver failure or hepatic fibrosis and cirrhosis and the rs599839 variant has been supported by UKBBC data. Notwithstanding, the rs599839 mutation was related to higher risk to develop severe HCC in NAFLD patients through a mechanism which was independent of the severity of fibrosis and the presence of cirrhosis. Hence, the mechanisms through which the variant oppositely impact on hepatic vs. cardiovascular disease differs from what we observed for the E167K TM6SF2 variant [10].

Therefore, in attempt to decipher the role of this *locus* in lipid handling, cardiovascular protection, and HCC predisposition, we demonstrated that patients who carry the rs599839 G allele showed higher hepatic mRNA levels of *SORT1*, *CELSR2* and *PSRC1*. It has also been previously reported that rs599839 variant strongly correlated with SORT1, PSRC1 and CELSR2 transcript levels in human liver [12,40,41], and we further reinforced this data with transcriptional studies and network analysis by STRING. In addition, the analysis of the expression of quantitative trait loci (eQTLs), confirmed the correlation between the rs599839 variant and hepato-specific PSRC1, SORT1 and CELSR2 expressions (Table S10). However, it remains unclear how the rs599839 genetic variation affects the expression of genes at this locus. Indeed, we did not find any transcription factor binding site in the proximity of the rs599839 polymorphism. Nonetheless, given the high linkage disequilibrium with the other 10 SNPs, we could speculate that they can impact on essential regulatory regions thus providing gene expression alterations. For instance, the rs12740374 SNP has been predicted to alter a binding site for CCAAT/enhancer-binding protein (C/EBP) transcription factors, resulting in a significantly increased SORT1 hepatic expression [18]. Notably, we demonstrated that the enhanced hepatic expression of SORT1 mainly affected circulating lipid profiles, lipoprotein turnover and release, while PSRC1 expression most strongly impacted on genes implicated in cell proliferation and survival thus possibly explaining the opposite effect of the rs599838 variation which protects against cardiovascular complications and as one predisposes to HCC, respectively. The rs599839 variant induces the expression of these genes whose effect is even more strong in homozygous status. Conversely, CELR2 expression neither impact on lipid levels nor proliferation.

Sortilin 1, encoded by *SORT1* gene, is directly involved in lipid metabolism and lipoprotein uptake [18,42]. It is a multi-ligand receptor mainly expressed in hepatocytes and macrophages, where it mediates the trafficking of diverse endogenous or exogenous proteins between the Trans-Golgi network and lysosomes, endosomes and plasma membranes [18,43]. As sorting receptor, sortilin 1 may regulate the hepatic expression of various genes, including LPL and APOE and lipid-related ones [44,45,46]. Specifically, hepatic sortilin 1 translocates apolipoproteins, mainly APOB to lysosomes for the autophagy-related degradation thus limiting VLDL/LDL formation and secretion and enhancing in turn their clearance. Thus, it mediates the reduction of circulating VLDL, TC and TG levels [47,48]. Similarly, sortilin 1 mediates the uptake of native LDL in macrophages for subsequent lysosomal hydrolysis [49]. We could speculate that the improved lipid profile in NAFLD patients carrying the rs599839 genetic variation may be explained by the enhanced LDL lysosomal degradation in hepatocytes and macrophages, due to the increased sortilin 1 expression.

Conversely, *PSRC1* gene encodes a proline-rich protein, that play a crucial role in mitosis by recruiting and regulating microtubule depolymerases (i.e., KIF2A) which destabilizes microtubules. *PSCR1* may act as oncogene by different mechanisms: it enhances β-catenin activation and cyclins production by binding to adenomatous polyposis coli 2 (APC2) and inhibits p53-binding protein 2 (ASPP2) [50]. It is targeted for regulation by the tumor suppressor protein p53, that is a well characterized transcription factor that mediates DNA repair, cell cycle arrest and apoptosis [17,51,52,53]. In particular, Hsieh and colleagues demonstrated that P53 suppresses the expression of both human PSRC1 mRNA and protein levels, specifically binding to a motif in *PSRC1* 5′ region [17].

Interestingly, in TCGA dataset the overexpression of PSRC1 has been strictly correlated with poor prognosis, tumor stage, advanced grade, and increased size of liver cancers, further reinforcing what we have observed in our cohort of NAFLD-HCC patients. Indeed, PSRC1 mRNA levels positively correlated with those of genes involved in cell proliferation and cell cycle progression, confirming our results from transcriptomic analyses. According to these findings, patients who overexpress PSRC1 showed an enhanced loss-of-function mutation rate in *TP53* gene, possibly explaining the absence of correlation between PSRC1 and TP53 expressions in HCC samples, as an escape from the TP53-mediated mechanisms of tumor suppression. Likewise, PSRC1 overexpression has been previously detected in tumor liver tissues and in hepatoma cells, where it is associated with HCC recurrence after resection [17]. Nonetheless, the mechanisms through which HCC tissues overexpress PSRC1, independently of the genetic background, remain to be fully elucidated and *TP53* mutations may partially explain this effect.

Overall, the evidence reported here points out PSRC1 as a possible novel target for HCC and the chr1p3.13 variation as a potentially useful marker to be incorporated in polygenic risk scores to differentiate the risk of progressive liver disease from that of cardiovascular events in individuals with dysmetabolism [54]. To this purpose, we tested whether the coexistence of *PNPLA3* rs738409, which is the most robust genetic predictor of advanced liver injuries, and the rs599839 in homozygosity could more strongly impact on HCC risk. We found that the co-presence of these two genetic mutations increased the HCC odd (OR: 8.1; 95% c.i. 2.22–29.40; *p* = 0.001), even after the adjustment for advanced fibrosis (OR: 8.77; 95% c.i. 4.48–17.17; *p* = 0.008) (data not shown). These findings may pave the way to introduce the combination of different variants in the management of NAFLD, although several studies are required to completely solve the riddle of the complex pathogenesis of progressive NAFLD in presence of environmental and genetic cues [55].

However, this study has some limitations. In UKKBC there is not individual data available about cancer history, thus precluding a formal evaluation of the interaction between *PSRC1* and the risk of HCC in this population. Moreover, the real proof-of-concept of the interaction between PSRC1 and TP53 is lacking.

In conclusion, the rs599839 variant is associated with protection against atherogenic dyslipidemia and with increased risk of HCC in NAFLD individuals. Several lines of evidence indicate that the mechanisms underlying these associations may involve the effect of the rs599839 variant on SORT1 and PSRC1 expressions, although prospective and functional studies are required to confirm this hypothesis and test the clinical relevance of these findings.

## Figures and Tables

**Table 1:** Demographic, anthropometric and clinical features of the Overall cohort (*n* = 1426) stratified according to enrollment criteria (*n* = 1295 Hepatology service cohort and *n* = 131 NAFLD-HCC).

	Overall Cohort (*n* = 1426)	Hepatology Service Cohort (*n* = 1295)	NAFLD-HCC (*n* = 131)	*p*-Value ^†^
Sex, M	770 (54)	669 (52)	101 (78)	<0.0001
Age, years	49.6 ± 13.6	48 ± 12.6	68 ± 10	<0.0001
BMI, kg/m^2^	34.2 ± 8.7	34.7 ± 8.76	28.7 ± 5.12	<0.0001
T2D, yes	385 (27)	310 (24)	75 (57)	<0.0001
Total cholesterol, mmol/L	5.1 ± 1.07	5.2 ± 1.05	4.2 ± 1.17	<0.0001
LDL cholesterol, mmol/L	3.1 ± 0.97	3.2 ± 0.95	2.37 ± 0.96	<0.0001
HDL cholesterol, mmoL/L	1.3 ± 0.39	1.3 ± 0.4	1.28 ± 0.51	0.73
Triglycerides, mmoL/L	1.37 {0.99–1.98}	1.4 {1.0–2.0}	1.18 {0.8–1.5}	0.11
ALT, IU/l	34 {20–57}	33 {20–57}	42 {27–55}	0.07
AST, IU/l	25 {19–38}	25 {18–37}	39 {26–59}	<0.0001
PNPLA3, I148M				<0.0001
I/I	557 (39)	524 (41)	33 (25)	
I/M	627 (44)	567 (43)	60 (46)	
M/M	242 (17)	204 (16)	38 (29)	
TM6SF2, E167K				0.25
E/E	1240 (87)	1131 (87.3)	109 (83.5)	
E/K	168 (11.8)	150 (11.6)	18 (14)	
K/K	18 (1.2)	14 (1.1)	4 (2.5)	
*MBOAT7,* rs641738 T allele				0.11
C/C	456 (32)	423 (33)	33 (25)	
C/T	642 (45)	581 (45)	61 (47)	
T/T	328 (23)	291 (22)	37 (28)	
rs599839 A>G				**0.01**
A/A	884 (62)	808 (63)	76 (58)	
A/G	456 (32)	417 (32)	39 (30)	
G/G	86 (6)	70 (5)	16 (12)	

**^†^** NAFLD-HCC vs. Hepatology service cohort.

**Table 2:** Demographic, anthropometric and clinical features of the overall cohort (*n* = 1426) stratified by rs599839 A>G genotype.

	AA (*n* = 884)	AG (*n* = 456)	GG (*n* = 86)	*p*-Value °	*p*-Value ^†^
Sex, M	487 (55)	234 (51)	49 (56)	0.33	0. 72
Age, years	49.18 ± 13.5	49.7 ± 13.5	50.0 ± 14.1	0.74	0.67
BMI, kg/m^2^	33.9 ± 8.50	34.4 ± 8.78	35.8 ± 10.4	0.16	0.10
IFG/T2D, yes (%)	234 (26)	1123 (26)	28 (32)	0.49	0.23
Glucose mg/dL	102 ± 30	103 ± 30	107 ± 39	0.56 *	0.18 *
HOMA-IR	5.3 ± 6.4	5.5 ± 11	4.56 ± 3.87	0.91 *	0.65 *
Insulin, IU/mL	20.7 ± 18.4	22.7 ± 34.5	15.9 ± 7.88	0.97 *	0.28 *
Total cholesterol, mmol/L	5.2 ± 1.1	5.0 ± 1.0	4.9 ± 1.0	0.005 *	0. 15 *
LDL cholesterol, mmol/L	3.25 ± 0.97	3.03 ± 0.95	2.8 ± 0.98	<0.0001 *	0.03 *
HDL cholesterol, mmol/L	1.26 ± 0.3	1.34 ± 0.43	1.36 ± 0.37	0.0003 *	0.12 *
Triglycerides, mmol/L	1.41 {1.0–2.0}	1.32 {0.92–1.97}	1.36 {1.02–1.97}	0.28 *	0.83 *
Dyslipidemia, yes (%)	300 (34)	127 (28)	15 (18)	0.01 *	0.007 *
ALT, IU/L	35 {21–56}	33 {20–62}	28 {19–52}	0.26 *	0.78 *
AST, IU/L	26 {19–38}	25 {19–39}	24{18–36}	0.76 *	0.72 *
Iron ug/dL	97.8 ± 44.4	96.8 ± 40.8	9.48 ± 33.6	0.23 **	0.67 **
Transferrin mg/dL	270.4 ± 60.8	264.6 ± 47.2	258.6 ± 47.0	0.13 **	0.48 **
Transferrin saturation (%)	30.1 ± 24.9	29.1 ± 15.2	34.9 ± 43.9	0.79 **	0.06 **
Ferritin ng/mL	324.4 ± 411.6	386.2 ± 508.2	459.2 ± 526.9	0.28 **	0.31 **

Values are reported as mean ± SD. number (%) or median {IQR}. as appropriate. BMI: body mass index. IFG: impaired fasting glucose. T2D: type 2 diabetes. Characteristics of participants were compared across the rs599839 genotypes using generalized linear model (for continuous characteristics) or nominal logistic regression model (for categorical characteristics). * Models were adjusted for gender, age, BMI, IFG/T2D, PNPLA3 I148M alleles. TM6SF2 E167K alleles and *MBOAT7* rs641738 T alleles. ** Models were adjusted also for HFE C282Y and HFE H63D alleles. ° Additive model, ^†^ Recessive model.

**Table 3:** Independent predictors of intima media thickness (IMT), plaque presence and hypertension in 1426 patients with NAFLD from the Overall cohort.

	IMT			Plaques, Yes			Hypertension, Yes		
	β	95% CI	*p*-Value	OR	95% CI	*p*-Value	OR	95% CI	*p*-Value
Sex, M	0.05	0.02–0.06	<0.0001	1.78	1.04–3.06	0.03	1.32	0.95–1.84	0.09
Age, years	0.008	0.006–0.009	<0.0001	1.09	1.07–1.12	<0.0001	1.06	1.05–1.07	<0.0001
BMI, kg/m^2^	0.001	−0.003–0.005	0.54	1.02	0.97–1.07	0.41	1.06	1.04–1.08	<0.0001
IFG/T2D, yes (%)	0.002	−0.02–0.02	0.86	1.54	0.91–2.61	0.11	1.68	1.18–2.38	0.003
TM6SF2, E167K allele	−0.02	−0.06–0.01	0.22	0.78	0.46–1.34	0.37	1.71	1.16–2.51	0.006
Statin use, yes	−0.01	−0.04–0.01	0.38	0.43	0.22–0.83	0.01	0.97	0.56–1.66	0.91
Active smoking	0.01	−0.01–0.03	0.36	1.840	1.08–3.14	0.02	1.30	0.92–1.84	0.12
rs599839 GG yes	−0.06	−0.10–0.02	**0.001**	0.22	0.05–0.92	**0.03**	0.43	0.18–0.98	**0.04**

CI: confidence interval. Values were obtained at multivariate generalized linear analysis (for IMT) or nominal logistic regression analysis (for Plaques and Hypertension) adjusted for sex, age, BMI (body mass index), T2D (type 2 diabetes mellitus) and TM6SF2 E167K alleles, statin use and active smoking by using a recessive model.

**Table 4:** Independent predictors of liver damage in 1426 patients with NAFLD from the Overall cohort.

	Steatosis			Lobular Inflammation			Ballooning			Fibrosis		
	β	95% CI	*p*-Value	β	95% CI	*p*-Value	β	95% CI	*p*-Value	β	95% CI	*p*-Value
Sex, M	0.29	0.17–0.41	<0.0001	0.25	0.13–0.37	<0.0001	0.29	0.14–0.44	<0.0001	0.32	0.20–0.44	<0.0001
Age, years	0.002	−0.007–0.01	0.67	0.02	0.01–0.03	<0.0001	0.03	0.02–0.04	<0.0001	0.07	0.04–0.06	<0.0001
BMI, kg/m^2^	0.04	0.02–0.05	<0.0001	0.03	0.01–0.04	<0.0001	−0.003	−0.02–0.01	0.67	0.002	−0.01–0.015	0.81
IFG/T2D, yes (%)	0.33	0.19–0.47	<0.0001	0.32	0.18–0.47	<0.0001	0.32	0.17–0.48	<0.0001	0.60	0.47–0.74	<0.0001
PNPLA3, I148M allele	0.43	0.28–0.57	<0.0001	0.35	0.19–0.50	<0.0001	0.28	0.10–0.47	0.002	0.49	0.34–0.64	<0.0001
TM6SF2, E167K allele	0.75	0.46–1.04	<0.0001	0.57	0.28–0.86	0.0001	0.41	0.08–0.75	0.01	0.57	0.29–0.84	<0.0001
*MBOAT7,* rs641738 T allele	0.11	−0.04–0.25	0.14	0.07	−0.08–0.22	0.33	0.02	−0.15–0.20	0.76	0.16	0.01–0.31	0.02
rs599839 G allele	−0.02	−0.19–0.16	0.83	−0.02	−0.21–0.16	0.80	0.20	−0.02–0.42	0.07	0.02	−0.16–0.19	0.82

CI: confidence interval. Values were obtained at multivariate ordinal regression analysis adjusted for sex, age, BMI (body mass index), T2D (type 2 diabetes mellitus) and PNPLA3 I148M alleles, TM6SF2 E167K alleles and *MBOAT7* rs641738 T allele by using an additive model.

**Table 5:** Independent predictors of NAFLD-HCC in 1426 patients with NAFLD (Cases *n* = 131).

	HCC °			HCC ^†^			HCC ° Adjusted for f > 2			HCC ^†^ Adjusted for f > 2		
	OR	95% CI	*p*-Value	OR	95% CI	*p*-Value	OR	95% CI	*p*-Value	OR	95% CI	*p*-Value
Sex, M	1.82	0.98–3.36	0.05	1.64	0.89–3.01	0.10	2.23	1.14–4.34	0.01	2.08	1.07–4.06	0.03
Age, years	1.16	1.12–1.20	<0.0001	1.16	1.12–1.20	<0.0001	1.13	1.09–1.17	<0.0001	1.13	1.09–1.17	<0.0001
BMI, kg/m^2^	0.87	0.82–0.93	<0.0001	0.88	0.83–0.93	<0.0001	0.90	0.84–0.96	0.0009	0.90	0.84–0.96	0.001
IFG/T2D, yes (%)	3.22	1.79–5.78	<0.0001	3.25	1.80–5.86	<0.0001	1.86	0.96–3.57	0.06	1.90	0.98–3.67	0.05
PNPLA3, I148M allele	1.35	0.93–1.95	0.11	1.34	0.92–1.94	0.12	1.02	0.67–1.53	0.93	1.01	0.67–1.52	0.95
TM6SF2, E167K allele	1.51	0.84–2.72	0.16	1.47	0.82–2.65	0.19	1.20	0.64–2.25	0.57	1.17	0.61–2.17	0.63
*MBOAT7,* rs641738 T allele	1.33	0.90–1.96	0.14	1.39	0.94–2.06	0.09	1.37	0.90–2.09	0.14	1.42	0.93–2.18	0.10
rs599839 G allele	1.70	1.08–2.70	**0.02**	5.85	2.12–16.12	**0.0006**	1.70	1.03–2.80	**0.03**	5.62	1.77–17.84	**0.003**

CI: confidence interval. Values were obtained at multivariate nominal logistic regression analysis adjusted for sex, age, BMI (body mass index), T2D (type 2 diabetes mellitus) and PNPLA3 I148M alleles, TM6SF2 E167K alleles and *MBOAT7* rs641738 T allele by using an additive ° or recessive model ^†^.

**Table 6:** Independent predictors of advance stage (S), tumor size (T) and Child-Pugh score in 131 NAFLD-HCC patients from the Overall cohort.

	Stage (S) > 1			Tumor size (T) > 1			Child-Pugh > A6		
	OR	95% CI	*p*-Value	OR	95% CI	*p*-Value	OR	95% CI	*p*-Value
Sex, M	1.10	0.33–3.65	0.87	0.82	0.234–2.83	0.01	0.47	0.08–2.90	0.42
Age, years	0.99	0.94–1.04	0.67	0.98	0.93–1.04	0.82	0.97	0.89–1.05	0.51
PNPLA3, I148M allele	0.61	0.30–1.23	0.16	0.41	0.19–0.85	0.93	0.43	0.13–1.45	0.17
TM6SF2, E167K allele	1.08	0.42–2.81	0.87	1.27	0.47–3.41	0.57	0.21	0.22–1.89	0.16
*MBOAT7*, rs641738 T allele	0.73	0.34–1.55	0.41	0.66	0.30–1.44	0.29	0.43	0.11–1.63	0.21
rs599839 G allele	3.27	1.36–7.85	**0.008**	2.86	1.17–6.96	**0.02**	4.38	1.35–14.22	**0.01**

CI: confidence interval. Values were obtained at multivariate nominal logistic regression analysis adjusted for sex, age, and PNPLA3 I148M alleles, TM6SF2 E167K alleles and *MBOAT7* rs641738 T allele by using an additive model.

**Table 7:** Association of the rs599839 A>G variant with liver-related outcomes and biochemical parameters in the UK Biobank cohort (UKBBC).

Phenotype	Cases	β	OR	*p*-Value
K70: Alcoholic liver disease	808	−1.99 × 10^−5^	1.01	0.86
K74: Fibrosis and cirrhosis of liver	805	3.31 × 10^−6^	0.99	0.97
K75: Other inflammatory liver diseases	662	−9.95 × 10^−5^	1.06	0.32
K76: Other diseases of liver	3351	−5.32 × 10^−6^	1	0.98
K70-K77: Diseases of liver	4894	−0.0002	1.02	0.39
High cholesterol	55,265	−0.017	1.17	7.82 × 10^−112^
E78 Disorders of lipoprotein metabolism and other lipidaemias	39,308	−0.010	1.14	4.3 × 10^−55^
E70-E90 Metabolic disorders	47,969	−0.010	1.11	3.09 × 10^−44^
I20-I25 Ischaemic heart diseases	33,387	−0.006	1.1	6.72 × 10^−25^
I25 Chronic ischaemic heart disease	27,772	−0.006	1.11	9.76 × 10^−25^
I20 Angina pectoris	19,935	−0.004	1.1	3.85 × 10^−17^
Heart/cardiac problem	32,474	−0.005	1.08	2.29 × 10^−16^
Angina	14,399	−0.003	1.12	2.88 × 10^−16^
Heart attack/myocardial infarction	10,356	−0.002	1.13	3.84 × 10^−14^
I21 Acute myocardial infarction	8764	−0.002	1.11	4.05 × 10^−9^
I30-I52 Other forms of heart disease	31,135	−0.002	1.04	0.0002
I50 Heart failure	5901	−0.001	1.08	0.0003
I71 Aortic aneurysm and dissection	1470	−0.0004	1.14	0.003
Pace-maker	1355	−0.0003	1.12	0.013
I10-I15 Hypertensive diseases	84,910	−0.002	1.01	0.018
G45 Transient cerebral ischaemic attacks and related syndromes	2765	−0.0004	1.07	0.03
I70 Atherosclerosis	1371	−0.0003	1.1	0.037

Biochemical parameters are assessed in the entire cohort (*n* = 500,000 subjects). HWE: 0.84 MAF: 0.23.

**Table 8:** Independent predictors of hepatic PSRC1 mRNA levels in 366 HCC patients from TCGA database.

	PSRC1 Expression		
	β	95% CI	*p*-Value
Sex, M	0.04	−0.08–0.31	0.50
Ethnicity; Hispanic	0.08	−0.18–0.35	0.55
Diagnosis Age	−0.0003	−0.01–0.006	0.50
Stage (S) >1	0.7	0.05–0.27	**0.006**
Sex, M	0.06	−0.06–0.18	0.31
Ethnicity; Hispanic	0.10	−0.16–0.36	0.45
Diagnosis Age	−0.001	−0.01–0.07	0.67
Grade (G) >2	0.17	0.05–0.29	**0.005**
Sex, M	0.05	−0.07–0.17	0.41
Ethnicity; Hispanic	0.08	−0.18–0.34	0.54
Diagnosis Age	−0.002	−0.01–0.006	0.53
Tumor size (T) >1	0.17	0.06–0.28	**0.003**

At multivariate generalized linear model adjusted for the confounders shown in the table.

## Data Availability

No new data were created or analyzed in this study. Data sharing is not applicable to this article.

## Supplementary Materials

The following are available online at https://www.mdpi.com/article/10.3390/cancers13081783/s1, Figure S1: The rs599839 variant impact on dyslipidemia but not on the histological spectrum of NAFLD; Figure S2: Genetic architecture of the region of 50.00 Kb in the 1p13.3 locus and LD of the rs599839 variant: Figure S3: PSRC1 and SORT1 correlation analyses; Figure S4: PSRC1 mRNA levels increases with tumor stage, grade and size; Figure S5: PSRC1 overexpressing patients up-regulate pathways mainly involved in cell proliferation and cell cycle progression; Figure S6: PSRC1 overexpressing patients down-regulate pathways mainly implicated in metabolic processes and fatty acid oxidation; Table S1: Genotypes and allele frequencies of the rs599839 A>G genotype of the Overall cohort (*n* = 1426) stratified according to enrollment criteria (*n* = 1295 Hepatology service cohort and *n* = 131 NAFLD-HCC); Table S2: Demographic, anthropometric and clinical features of 125 obese patients (Transcriptomic cohort), of whom RNA samples were available for RNAseq analysis; Table S3: Associations between the rs599839 genetic variant and the spectrum of liver damage in patients from the Overall cohort (*n* = 1426), ^†^ at recessive mode; Table S4: Independent predictors of cirrhosis in the Overall cohort (*n* = 1426); Table S5: LD analysis across the region spanning 50.00 Kb (chr1: 109,800,000–109,850,000; Human (GRCh37.p13)) and rs599839 variant; Table S6: Correlation analysis of gene expression of PSRC1 and genes involved in lipid synthesis, cell proliferation and stemness markers in HCC samples (*n* = 366) of TCGA database; Table S7: REACTOME pathway-enriched analysis for 6353 genes co-regulated with PSRC1 by Toppgene in 366 HCC from TCGA dataset; Table S8: REACTOME pathway-enriched analysis for 2129 genes up-regulated in patients with PSRC1 altered expression by Toppgene; Table S9: REACTOME pathway-enriched analysis for 1665 genes down-regulated in patients with PSRC1 altered expression by Toppgene; Table S10: Analysis of expression of quantitative trait loci (eQTLs) denoting correlations between rs599839 and liver-specific gene expression levels.

## Abbreviation

APOB, Apolipoprotein B; BMI, body mass index; CAD, coronary artery disease; CCN, cyclin; CDK, cyclin dependent kinases; CELSR2, Cadherin EGF LAG Seven-Pass G-Type Receptor 2; DGAT2, Diacylglycerol O-acyltransferase 2; FDR, False Discovery Rate; IGT, impaired glucose tolerance; GWAS, genome wide association study; HDL, high-density lipoprotein; HCC, hepatocellular carcinoma; IMT, intima-media thickness; LDL, low-density lipoprotein; LPL, Lipoprotein lipase; MBOAT7, Membrane Bound O-Acyltransferase Domain Containing 7; MTTP, Microsomal Triglyceride Transfer Protein; NAFLD, nonalcoholic fatty liver disease; NASH, nonalcoholic steatohepatitis; PCNA, Proliferating cell nuclear antigen; PNPLA3, Patatin-like Phospholipase domain-containing 3; PSRC1, Proline And Serine Rich Coiled-Coil 1; SREBP1, Sterol Regulatory Element-binding Protein-1; SORT1, sortilin-1; TCGA-LIHC, The Cancer Genome Atlas-Liver Hepatocellular Carcinoma; TM6SF2, Transmembrane 6 Superfamily member gene 2; TP53, Tumor protein p53; T2D, type 2 diabetes mellitus; UKBBC, UK Biobank Cohort.
