# Supplementary material for: The rs599839 A>G Variant Disentangles Cardiovascular Risk and Hepatocellular Carcinoma in NAFLD Patients
Source: Cancers (Basel). 2021 Apr 8;13(8):1783. doi: 10.3390/cancers13081783 (PMC8068289; doi:10.3390/cancers13081783)
Supplement: Supplementary file 1 [file cancers-13-01783-s001.pdf]

cancers

Article

The rs599839 A>G Variant Disentangles Cardiovascular Risk and Hepatocellular Carcinoma in NAFLD Patients

Marica Meroni [1,2], Miriam Longo [1,3], Erika Paolini [1,4], Anna Alisi [5], Luca Miele [6], Emilia Rita De Caro [1], Giuseppina Pisano [1], Marco Maggioni [7], Giorgio Soardo [8], Luca Vittorio Valenti [2,9], Anna Ludovica Fracanzani [1,2] and Paola Dongiovanni [1,*]

[1] General Medicine and Metabolic Diseases, Fondazione IRCCS Ca' Granda Ospedale Maggiore Policlinico, 20122 Milano, Italy; marica.meroni@unimi.it (M.M.); miriam.longo@unimi.it (M.L.); erika.paolini@unimi.it (E.P.); emilia.decaro@policlinico.mi.it (E.R.D.C.); Giuseppina.pisano@policlinico.mi.it (G.P.); anna.fracanzani@unimi.it (A.L.F.)
[2] Department of Pathophysiology and Transplantation, Università degli Studi di Milano, 20122 Milano, Italy; luca.valenti@unimi.it
[3] Department of Clinical Sciences and Community Health, Università degli Studi di Milano, 20122 Milano, Italy
[4] Department of Pharmacological and Biomolecular Sciences, Università degli Studi di Milano, 20133 Milano, Italy
[5] Research Unit of Molecular Genetics of Complex Phenotypes, Bambino Gesù Children Hospital, IRCCS, 00165 Rome, Italy; anna.alisi@opbg.net
[6] Area Medicina Interna, Gastroenterologia e Oncologia Medica, Fondazione Policlinico Universitario A. Gemelli IRCCS, 00168 Rome, Italy; luca.miele@policlinicogemelli.it
[7] Department of Pathology, Fondazione IRCCS Ca' Granda Ospedale Maggiore Policlinico, 20122 Milano, Italy; marco.maggioni@policlinico.mi.it
[8] Clinic of Internal Medicine-Liver Unit Department of Medical Area (DAME), University School of Medicine, Udine, Italy and Italian Liver Foundation AREA Science Park—Basovizza Campus, 34149 Trieste, Italy; giorgio.soardo@asuiud.sanita.fvg.it
[9] Precision Medicine, Department of Transfusion Medicine and Hematology, Fondazione IRCCS Ca' Granda Ospedale Maggiore Policlinico, 20122 Milano, Italy
* Correspondence: paola.dongiovanni@policlinico.mi.it; Tel.: +39-02-5503-3467; Fax: +39-02-5503-4229

1. Transcriptomic analysis

Gene expression was performed in a subset of 125 severely obese patients (21 without and 104 with NAFLD) (Transcriptomic cohort) belonging to the Hepatology service cohort, of whom percutaneous liver biopsy was performed during bariatric surgery at Fondazione IRCCS Cà Granda, Ospedale Policlinico, Milan, Italy. The study was conformed to the Declaration of Helsinki and approved by the Institutional Review Boards and their Ethics Committees. All participants gave written informed consent. Clinical characteristics of the Transcriptomic cohort are presented in Table S2.

Total RNA was isolated using RNeasy mini-kit (Qiagen, Hulsterweg, Germany), according to the manufacturer's instructions. RNA quality was assessed through Agilent 2100 Bioanalyzer and samples with RNA integrity numbers (RIN) greater than or equal to 7 were used for library preparation. RNA sequencing was performed in paired-end mode with a read length of 150nt using the Illumina HiSeq 4000 (Novogene, Hong Kong, China). Illumina raw reads were mapped against the Human Genome [1] using a custom pipeline based on the standard primary analysis procedure. The pipeline performed the primary analysis step including FASTQ quality check (FastQC software, Babraham Bioinformatics, Cambridge, UK), low-quality reads trimming with Trimmomatic [2] and mapping on GRCh37 reference genome using STAR mapper [3]. RNASeq quality control was performed, and samples with less than 10 million reads uniquely mapped or with less than 60% uniquely mapped/mapped reads were excluded from the analysis. Gene reads count was performed using RSEM software [4]. To quantify gene expression RSEM per-gene counts were normalized using DESeq2 package [5].

2. Tumor stage system classification

The stage system comprises the overall parameters that describes the tumor (primary tumor size, spreading into lymph-nodes and metastasis. Higher stage was used to define an enhanced size and number of the tumor and its spreading in nearby tissues and distant part of the body: S0: carcinoma in situ; S1-4: advanced carcinoma progressively associated to poor prognosis (Stage 1 have often a good prognosis).

Depending on the amount of abnormality, HCC were graded as: G1: well differentiated (low grade); the organization of tumor tissue appears close to normal, G2: moderately differentiated (intermediate grade), G3: poorly differentiated (high grade), G4: undifferentiated (high grade).

Tumor size (T) and/or extension defines the aspects of original primary tumor, such as size, growth and invasion to nearby tissues: T0: no evidence of primary tumor, T1-4: increasing tumor size and spreading.

3. Bioinformatic Resources

LD was calculated based on the 1000 genomes project population (n = 503 samples) using LD calculator from ensemble GRCh37 (https://grch37.ensembl.org/Homo_sapiens/Tools/LD).

LD values were calculated by a pairwise estimation between SNPs genotyped in the same samples and within a given window of 50.00 Kb spanning the chr1: 109,800,000-109,850,000. An established method was used to estimate the maximum likelihood of the proportion that each possible haplotype contributed to the double heterozygote. R squared and D-prime calculation were based on maximum likelihood solution to the cubic equation. We reported those variants whose R-square ranged from 0.8 to 1, which we defined as indicative of the presence of strong LD.

To constitute a protein-protein interaction network between PSRC1, SORT1 and CELSR2, we performed a prediction analysis by using Search Tool for the Retrieval of Interacting Genes/Proteins (STRING) (https://string-db.org/; version 11.0).

To clarify biological functions of genes co-regulated with PSCR1 in TCGA dataset we performed a pathway-enriched analyses on the most strictly correlated genes with PSRC1 (q-Value < 0.01 at Benjamini correction) by using Toppgene (https://toppgene.cchmc.org/). The same bioinformatic tool has been used to investigate which pathways are enriched among the differentially expressed genes, identified in the group of patients with altered expression of PSRC1.

References

1. Cunningham, F.; Amode, M.R.; Barrell, D.; Beal, K.; Billis, K.; Brent, S.; Carvalho-Silva, D.; Clapham, P.; Coates, G.; Fitzgerald, S.; et al. Ensembl 2015. *Nucleic Acids Res.* **2014**, *43*, D662-D669.
2. Bolger, A.M.; Lohse, M.; Usadel, B. Trimmomatic: a flexible trimmer for Illumina sequence data. *Bioinformatics*. **2014**, *30*, 2114-2120.
3. Dobin, A.; Davis, C.A.; Schlesinger, F.; Drenkow, J.; Zaleski, C.; Jha, S.; Batut, P.; Chaisson, M.; Gingeras, T.R. STAR: ultrafast universal RNA-seq aligner. *Bioinformatics* **2013**, *29*, 15-21
4. Li, B.; Dewey, C.N. RSEM: accurate transcript quantification from RNA-Seq data with or without a reference genome. *BMC Bioinformatics*, **2011**, *12*, 323. doi: 10.1186/1471-2105-12-323.
5. Love, M.I.; Huber, W.; Anders, S. Moderated estimation of fold change and dispersion for RNA-seq data with DESeq2. *Genome. Biol.*, **2014**, *15*, 550. doi: 10.1186/s13059-014-0550-8.

Table S1. Genotypes and allele frequencies of the rs599839 A>G genotype of the Overall cohort (*n* = 1426) stratified according to enrollment criteria (*n* = 1295 Hepatology service cohort and *n* = 131 NAFLD-HCC).

	**1000G (*n* = 404)**	**Overall (*n* = 1426)**	**Hepatology Service Cohort (*n* = 1295)**	**NAFLD-HCC (*n* = 131)**
**AA (%)**	242 (60)	884 (62)	808 (63)	76 (58)
**AG (%)**	135 (33.4)	456 (32)	417 (32)	39 (30)
**GG (%)**	27 (6.7)	86 (6)	70 (5)	16 (12)
**Total**	404	1426	1295	131
**A (%)**	619 (76.6)	2224 (78)	2033 (79)	191 (73)
**G (%)**	189 (23.4)	628 (22)	557 (21)	71 (27)
**Total**	808	2852	2590	262

1000G (European Non-Finnish) is consistent with Hardy-Weinberg equilibrium (p = 0.17). Overall Cohort is not consistent with Hardy-Weinberg equilibrium (p = 0.01). Hepatology service cohort is consistent with Hardy-Weinberg equilibrium (p = 0.09). NAFLD-HCC is not consistent with Hardy-Weinberg equilibrium (p = 0.004). Fisher exact 1000G *vs* Hepatology service cohort: 0.26. Fisher exact 1000G vs. NAFLD-HCC: 0.24. Fisher exact NAFLD-HCC *vs* Hepatology service cohort: 0.03.

Table S2. Demographic, anthropometric and clinical features of 125 obese patients (Transcriptomic cohort), of whom RNA samples were available for RNAseq analysis.

	**Normal Liver (*n* = 21)**	**Simple Steatosis (*n* = 73)**	**Severe NAFLD (*n* = 31)**	***p*-Value**
Sex. F	21 (91)	77 (90)	26 (62)	0.05
Age, years	41 ± 2.0	43 ± 1.1	44 ± 1.6	0.43
BMI, kg/m^2	37 ± 6.7	41 ± 6.4	44 ± 8.0	0.001
IFG/T2D, yes	1 (4.5)	10 (12.2)	6 (15)	0.4
Total cholesterol, mg/dL	193.4 ± 54.1	216.5 ± 38.6	193.3 ± 42.5	0.06
LDL cholesterol, mg/dL	135.3 ± 34.8	135.3 ± 30.9	119.9 ± 42.5	0.13
HDL cholesterol, mg/dL	58 ± 11.6	54.1 ± 11.6	50.3 ± 15.5	0.08
Triglycerides, mg/dL	104.9 ± 43.7	131.2 ± 61.2	139.9 ± 78.7	0.22
ALT, IU/L	16 {13-21}	19 {15-26}	31 {24-94}	0.0001
AST, IU/L	16 {13-19}	17 {15-21}	24 {18-31}	0.009
PNPLA3, 148M allele, yes	11 (50)	40 (47)	27 (64)	0.2
TM6SF2, 167K allele, yes	2 (9)	7 (8)	4 (10)	0.9
MBOAT7, T allele, yes	13 (56)	49 (59)	31 (73)	0.2

Values are reported as mean ± SD. number (%) or median {IQR}. as appropriate. BMI: body mass index; IFG: impaired fasting; T2D: type 2 diabetes mellitus**.**

Table S3. Associations between the rs599839 genetic variant and the spectrum of liver damage in patients from the Overall cohort (*n* = 1426), ┼ at recessive model.

	**Steatosis┼**			**Necroinflammation┼**			**Ballooning ┼**			**Fibrosis ┼**		
	**β**	**95% CI**	***p*-Value**	**β**	**95% CI**	***p*-Value**	**β**	**95% CI**	***p*-Value**	**β**	**95% CI**	***p*-Value**
Sex, M	0.29	0.17-0.41	<0.0001	0.25	0.13-0.37	<0.0001	0.28	0.14-0.43	0.0001	0.31	0.19-0.43	<0.0001
Age, years	0.002	-0.007-0.01	0.69	0.02	0.01-0.03	<0.0001	0.03	0.02-0.04	<0.0001	0.05	0.04-0.06	<0.0001
BMI, kg/m^2	0.04	0.03-0.05	<0.0001	0.03	0.01-0.05	<0.0001	-0.003	-0.02-0.01	0.65	0.001	-0.01-0.015	0.87
IFG/T2D, yes (%)	0.33	0.19-0.47	<0.0001	0.33	018-0.46	<0.0001	0.32	0.17-0.47	<0.0001	0.60	0.46-0.74	<0.0001
PNPLA3, I148M allele	0.43	0.27-0.57	<0.0001	0.35	0.19-0.50	<0.0001	0.28	0.10-0.47	0.002	0.49	0.34-0.65	<0.0001
TM6SF2, E167K allele	0.74	0.46-1.03	<0.0001	0.57	0.28-0.86	0.0001	0.41	0.07-0.73	0.01	0.58	0.30-0.85	0.0001
MBOAT7, rs641738 T allele	0.11	-0.04-0.25	0.14	0.07	-0.08-0.22	0.33	0.03	-0.15-0.21	0.73	0.17	0.019-0.31	0.03
rs599839 G allele	-0.12	-0.35-0.11	0.32	0.07	-0.18-0.32	0.58	0.21	-0.07-0.49	0.12	0.21	-0.02-0.44	**0.08**

CI: confidence interval. Values were obtained at multivariate ordinal regression analysis adjusted for sex, age, BMI (body mass index), T2DM (type 2 diabetes mellitus) and PNPLA3 I148M alleles, TM6SF2 E167K alleles and *MBOAT7* rs641738 T allele.

Table S4. Independent predictors of cirrhosis in the Overall cohort (*n* = 1426).

	Cirrhosis°			Cirrhosis†		
	**OR**	**95% CI**	***p*-Value**	**OR**	**95% CI**	***p*-Value**
Sex, M	0.97	0.89-1.55	0.89	0.95	0.59-1.50	0.80
Age, years	1.11	1.09-1.14	<0.0001	1.11	1.09-1.14	<0.0001
BMI, kg/m^2	0.95	0.91-0.98	0.005	0.95	0.91-0.98	0.004
IFG/T2D, yes (%)	3.92	2.46-6.23	<0.0001	3.92	2.47-6.247	<0.0001
PNPLA3, I148M allele	1.79	1.33-2.41	0.0001	1.79	1.33-2.41	0.0004
TM6SF2, E167K allele	1.571	0.96-2.59	0.07	1.57	0.95-2.58	0.06
MBOAT7, rs641738 T allele	1.06	0.79-1.44	0.51	1.08	0.79-1.46	0.47
rs599839 G allele	1.17	0.80-1.70	0.40	1.90	0.77-4.66	0.12

OR: odds ratio; CI: confidence interval. Values were obtained at multivariate nominal logistic regression analysis adjusted for sex, age, BMI (body mass index), T2DM (type 2 diabetes mellitus) and PNPLA3 I148M alleles, TM6SF2 E167K alleles and *MBOAT7* rs641738 T allele. °Additive model; † Recessive model.

Table S5. LD analysis across the region spanning 50.00 Kb (chr1: 109,800,000-109,850,000; Human (GRCh37.p13)) and rs599839 variant.

SNP	Phenotype in UKBBC*	β; *p*-Value	Location	Consequence	MAF	R Squared	D′
rs1277930 A>G	High Cholesterol	-0.017; 4.4×10^{-114}	1:109822143	Intergenic variant	0.36	1	1
rs583104 T>G	High Cholesterol	-0.017; 2.9×10^{-115}	1:109821307	Intergenic variant	0.36	1	1
rs4970836 A>G	High Cholesterol	-0.017; 9.0×10^{-115}	1:109821797	Intergenic variant	0.35	0.95	1
rs602633 G>T	High Cholesterol	-0.017; 3.3×10^{-109}	1:109821511	Intergenic variant	0.35	0.93	1
rs7528419 A>G	High Cholesterol	-0.017; 6.2×10^{-117}	1:109817192	3′ UTR variant	0.20	0.91	0.999
rs629301 T>G	High Cholesterol	-0.017; 7.5×10^{-120}	1:109818306	3′ UTR variant	0.23	0.91	0.999
rs646776 T>C	High Cholesterol	-0.017; 2.1×10^{-119}	1:109818530	Intergenic variant	0.23	0.91	0.999
rs12740374 G>T	High Cholesterol	-0.02; 2.7×10^{-122}	1:109817590	3′ UTR variant	0.19	0.91	0.999
rs3832016 T>(-)	NA	NA NA	1:109818159	3′ UTR variant	0.23	0.86	1
rs660240 C>T	High Cholesterol	-0.017; 4.6×10^{-116}	1:109817838	3′ UTR variant	0.23	0.86	1

Single nucleotide variants significantly correlated with rs599839 (chr1: 1:109822166) in a range of 50.00Kb genomic region (R squared from 0.8 to 1). NA, Not applicable. * Most significantly related phenotype to the variant. Estimate beta refers to the minor allele presence. MAF: global minor allele frequency.

Table S6. Correlation analysis of gene expression of PSRC1 and genes involved in lipid synthesis, cell proliferation and stemness markers in HCC samples (*n* = 366) of TCGA database.

mRNA	PSRC1 Expression		
	**ρ**	***p-Value***	***q*-Value**
APOB	-0.40	8.3×10^{-16}	2.8×10^{-14}
SORT1	0.37	2.3×10^{-13}	5.2×10^{-12}
CELSR2	0.22	2.3×10^{-5}	9.7×10^{-5}
DGAT2	-0.13	0.009	0.02
WNT3A	0.12	0.02	0.04
WNT7B	0.15	0.003	0.008
WNT10A	0.25	9.9×10^{-7}	5.7×10^{-6}
WNT10B	0.19	0.0002	0.0007
WNT16	0.12	0.014	0.029

TERT	0.33	7.2 x 10^{-11}	9.8 x 10^{-10}
RAD21	0.26	4.2 x 10^{-7}	2.6 x 10^{-6}
CCNB1	0.65	3.9 x 10^{-46}	3.2 x 10^{-43}
CCNB2	0.67	1.1 x 10^{-49}	1.8 x 10^{-46}
CCNE1	0.46	4.1 x 10^{-21}	2.7 x 10^{-19}
CCNE2	0.41	2.2 x 10^{-16}	8.1 x 10^{-15}
CCNF	0.67	1.2 x 10^{-49}	1.8 x 10^{-46}
CCNI2	0.33	7.9 x 10^{-11}	1.07 x 10^{-9}
CDK1	0.65	7.8 x 10^{-46}	5.7 x 10^{-43}
CDK2	0.44	2.8 x 10^{-19}	1.5 x 10^{-17}
CDK4	0.56	1.4 x 10^{-32}	2.2 x 10^{-30}
E2F2	0.61	2.6 x 10^{-39}	7.6 x 10^{-37}
PCNA	0.50	1.2 x 10^{-24}	1.2 x 10^{-22}
MKI67	0.57	1.4 x 10^{-33}	2.6 x 10^{-31}
CDC20	0.72	2.4 x 10^{-60}	5.0 x 10^{-56}
MCM2	0.62	1.8 x 10^{-40}	6.2 x 10^{-38}
MCM3	0.54	2.0 x 10^{-29}	2.5 x 10^{-27}
MCM4	0.43	3.6 x 10^{-18}	1.7 x 10^{-16}
MCM5	0.62	1.7 x 10^{-40}	5.8 x 10^{-38}
MCM6	0.56	1.8 x 10^{-32}	2.9 x 10^{-30}
MCM7	0.59	4.5 x 10^{-36}	1.0 x 10^{-33}
MCM8	0.43	2.8 x 10^{-18}	1.3 x 10^{-16}
MCM9	0.12	0.01	0.036
MCM10	0.62	2.3 x 10^{-40}	7.3 x 10^{-38}
TP53	0.06	0.27	0.36

ϱ: Spearman's rank correlation coefficient; *q*-Value is derived from Benjamini-Hochberg FDR correction procedure.

Table S7. REACTOME pathway-enriched analysis for 6353 genes co-regulated with PSRC1 by Toppgene in 366 HCC from TCGA dataset**.**

REACTOME Term	Count	*p-Value*	*q*-Value
**Cell Cycle**	**624**	**3.00** x 10^{-23}	**1.07** x 10^{-19}
Mitotic Prometaphase	111	1.51 x 10^{-18}	2.70 x 10^{-15}
Cell Cycle, Mitotic	517	7.43 x 10^{-18}	8.86 x 10^{-15}
RHO GTPases Activate Formins	117	2.03 x 10^{-16}	1.73 x 10^{-13}
Resolution of Sister Chromatid Cohesion	103	2.42 x 10^{-16}	1.73 x 10^{-13}
Processing of Capped Intron-Containing Pre-mRNA	248	1.12 x 10^{-14}	6.68 x 10^{-12}
Valine, leucine and isoleucine degradation	48	5.95 x 10^{-14}	3.04 x 10^{-11}
DNA strand elongation	32	2.16 x 10^{-12}	9.65 x 10^{-10}
Fatty acid metabolism	46	4.34 x 10^{-12}	1.75 x 10^{-9}
Spliceosome	134	1.01 x 10^{-11}	3.61 x 10^{-9}
mRNA Splicing-Major Pathway	188	1.22 x 10^{-11}	3.98 x 10^{-9}
Valine, Leucine and Isoleucine Degradation	30	1.90 x 10^{-11}	5.66 x 10^{-9}
mRNA Splicing	196	4.54 x 10^{-11}	1.24 x 10^{-8}
Mitotic Metaphase and Anaphase	182	5.49 x 10^{-11}	1.40 x 10^{-8}
Mitotic Anaphase	181	9.80 x 10^{-11}	2.33 x 10^{-8}
Metabolism of amino acids and derivatives	367	1.34 x 10^{-10}	3.00 x 10^{-8}
Metabolism of lipids and lipoproteins	816	1.5 x 10^{-10}	3.28 x 10^{-8}
Gene Expression	1844	3.29 x 10^{-10}	6.35 x 10^{-8}
Activation of ATR in response to replication stress	37	3.37 x 10^{-10}	6.35 x 10^{-8}
Biological oxidations	229	3.95 x 10^{-10}	7.06 x 10^{-8}
DNA replication	36	8.99 x 10^{-10}	1.53 x 10^{-7}
E2F transcription factor network	73	2.84 x 10^{-9}	3.76x 10^{-7}
Extension of Telomeres	31	1.30 x 10^{-8}	1.61 x 10^{-6}

q-Value is derived from Benjamini-Hochberg FDR correction procedure.

Table S8. REACTOME pathway-enriched analysis for 2129 genes up-regulated in patients with PSRC1 altered expression by Toppgene.

REACTOME Term	Count	*p-Valu10*	*q*-Valu10
Cell Cycle	242	6.80×10^{-81}	1.65×10^{-77}
Gene Expression	442	1.12×10^{-72}	1.37×10^{-69}
Cell Cycle, Mitotic	204	3.68×10^{-69}	2.99×10^{-66}
Processing of Capped Intron-Containing Pre-mRNA	128	2.01×10^{-59}	1.22×10^{-56}
mRNA Splicing	102	8.16×10^{-48}	3.98×10^{-45}
mRNA Splicing - Major Pathway	99	4.89×10^{-47}	1.98×10^{-44}
Spliceosome	79	1.40×10^{-42}	4.88×10^{-40}
Mitotic Prometaphase	62	9.45×10^{-32}	2.88×10^{-29}
Resolution of Sister Chromatid Cohesion	56	5.70×10^{-28}	1.5×10^{-25}
M Phase	103	3.01×10^{-27}	7.33×10^{-25}
RHO GTPases Activate Formins	59	4.37×10^{-27}	9.69×10^{-25}
Mitotic Metaphase and Anaphase	74	3.93×10^{-26}	7.98×10^{-24}
Cell Cycle Checkpoints	68	1.64×10^{-18}	1.60×10^{-16}
Transcriptional Regulation by TP53	99	2.91×10^{-18}	2.73×10^{-16}
Mitotic G1-G1/S phases	55	4.38×10^{-18}	3.96×10^{-16}
DNA Replication	47	6.72×10^{-18}	5.85×10^{-16}
Mitotic G2-G2/M phases	62	5.48×10^{-17}	4.45×10^{-15}
Anchoring of the basal body to the plasma membrane	43	8.65×10^{-17}	6.8×10^{-15}
G2/M Transition	61	1.32×10^{-16}	1.0×10^{-14}
Extension of Telomeres	23	1.36×10^{-16}	1.0×10^{-14}
S Phase	50	1.57×10^{-16}	1.12×10^{-14}
Regulation of TP53 Activity	55	1.31×10^{-15}	8.65×10^{-14}

q-Value is derived from Benjamini-Hochberg FDR correction procedure.

Table S9. REACTOME pathway-enriched analysis for 1665 genes down-regulated in patients with PSRC1 altered expression by Toppgene.

REACTOME Term	Count	*p-Value*	*q*-value
Metabolic pathways	239	5.09×10^{-29}	1.40×10^{-25}
Fatty acid degradation	29	4.89×10^{-20}	6.77×10^{-17}
Valine, leucine and isoleucine degradation	30	1.07×10^{-19}	9.88×10^{-17}
Metabolism of lipids and lipoproteins	156	1.96×10^{-19}	1.35×10^{-16}
MAP00071 Fatty acid metabolism	29	3.10×10^{-19}	1.71×10^{-16}
MAP00280 Valine leucine and isoleucine degradation	21	7.44×10^{-18}	3.43×10^{-15}
Valine, Leucine and Isoleucine Degradation	22	4.59×10^{-17}	1.81×10^{-14}
Complement and coagulation cascades	35	2.65×10^{-16}	9.19×10^{-14}
Peroxisome	33	8.61×10^{-14}	2.64×10^{-11}
Biological oxidations	59	1.89×10^{-13}	1.89×10^{-13}
Mitochondrial Fatty Acid Beta-Oxidation	16	5.25×10^{-11}	5.25×10^{-11}
Fatty acid Metabolism	12	1.20×10^{-12}	1.20×10^{-12}
Peroxisomal lipid metabolism	17	3.03×10^{-10}	3.03×10^{-10}
PPAR signaling pathway	26	4.60×10^{-10}	6.07×10^{-8}
beta-Oxidation	10	2.54×10^{-9}	2.61×10^{-7}
Fatty acid, triacylglycerol, and ketone body metabolism	54	3.93×10^{-9}	3.88×10^{-7}
Complement cascade	21	4.61×10^{-9}	4.39×10^{-7}
fatty acid beta-oxidation (peroxisome)	10	1.01×10^{-8}	9.33×10^{-7}
MAP00650 Butanoate metabolism	12	1.05×10^{-8}	9.43×10^{-7}
MAP00310 Lysine degradation	11	1.19×10^{-8}	1.03×10^{-6}

q-Value is derived from Benjamini-Hochberg FDR correction procedure.

Table S10. Analysis of expression of quantitative trait loci (eQTLs) denoting correlations between rs599839 and liver-specific gene expression levels.

Query SNP	Gene	Association	NES
rs599839	*SORT1*	5.2×10^{-32}	1.1
rs599839	*PSRC1*	6.5×10^{-29}	1.1
rs599839	*CELSR2*	2.4×10^{-23}	0.90
rs599839	*SYPL2*	0.0000038	0.48
rs599839	*ATXN7L2*	0.0000087	0.36

Data was extracted from the integrated eQTL database in March 2020 and it is available at: https://www.gtexportal.org/. Query was specifically done on rs599839. NES, normalized effect size using G allele as reference.

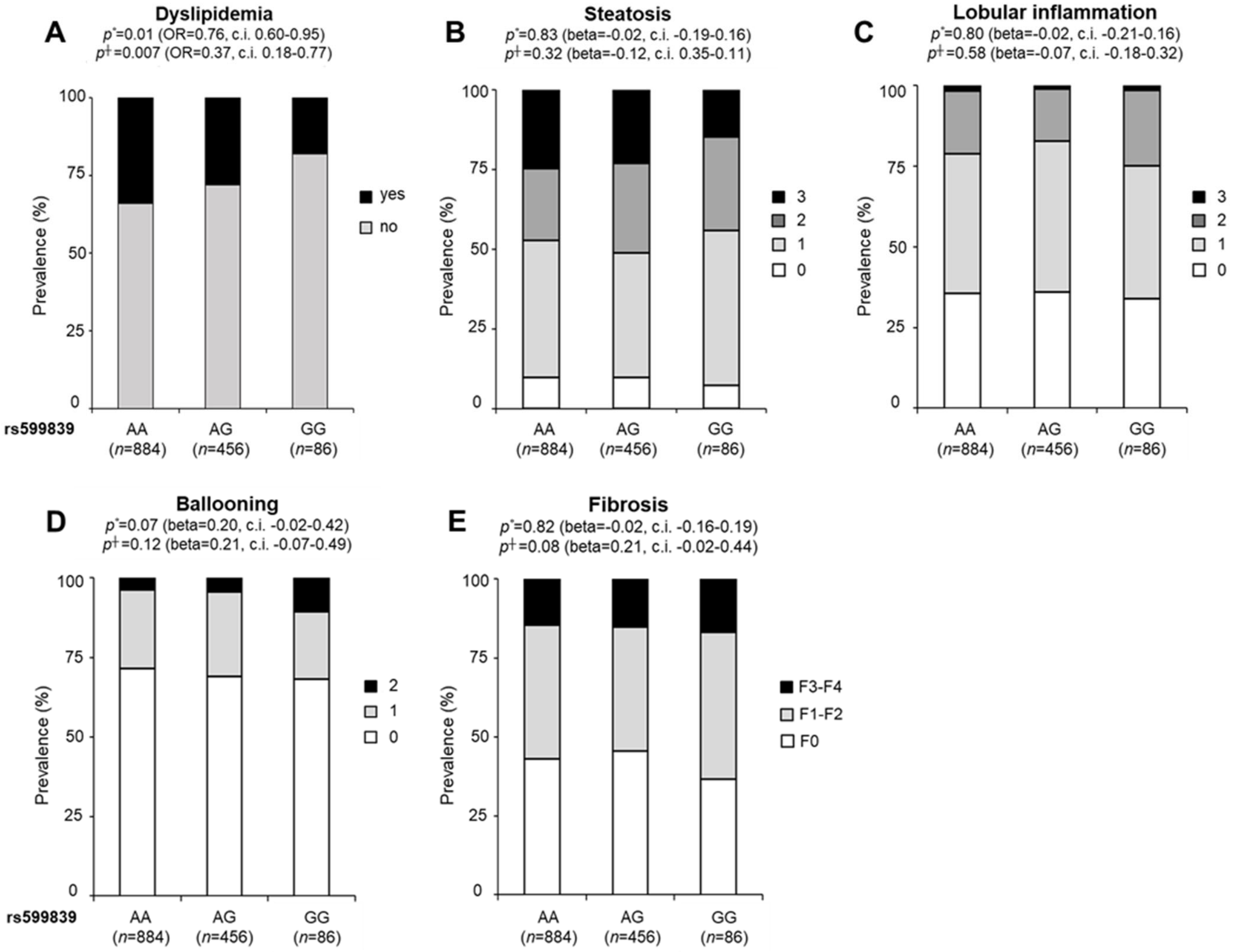

Figure S1. The rs599839 variant impact on dyslipidemia but not on the histological spectrum of NAFLD. Association of the rs599839 variant with dyslipidemia (**A**), steatosis (**B**), lobular inflammation (**C**), fibrosis stage (**D**) and ballooning grade (**E**), in NAFLD patients from the Overall cohort (*n* = 1426).Multivariable nominal logistic regression analysis (for Dyslipidemia) or ordinal regression analysis (for steatosis, lobular inflammation, fibrosis stage and ballooning grade), adjusted for age, sex, BMI, T2D, presence of PNPLA3 I148M, TM6SF2 E167K and MBOAT7 T alleles at °additive or †recessive model.

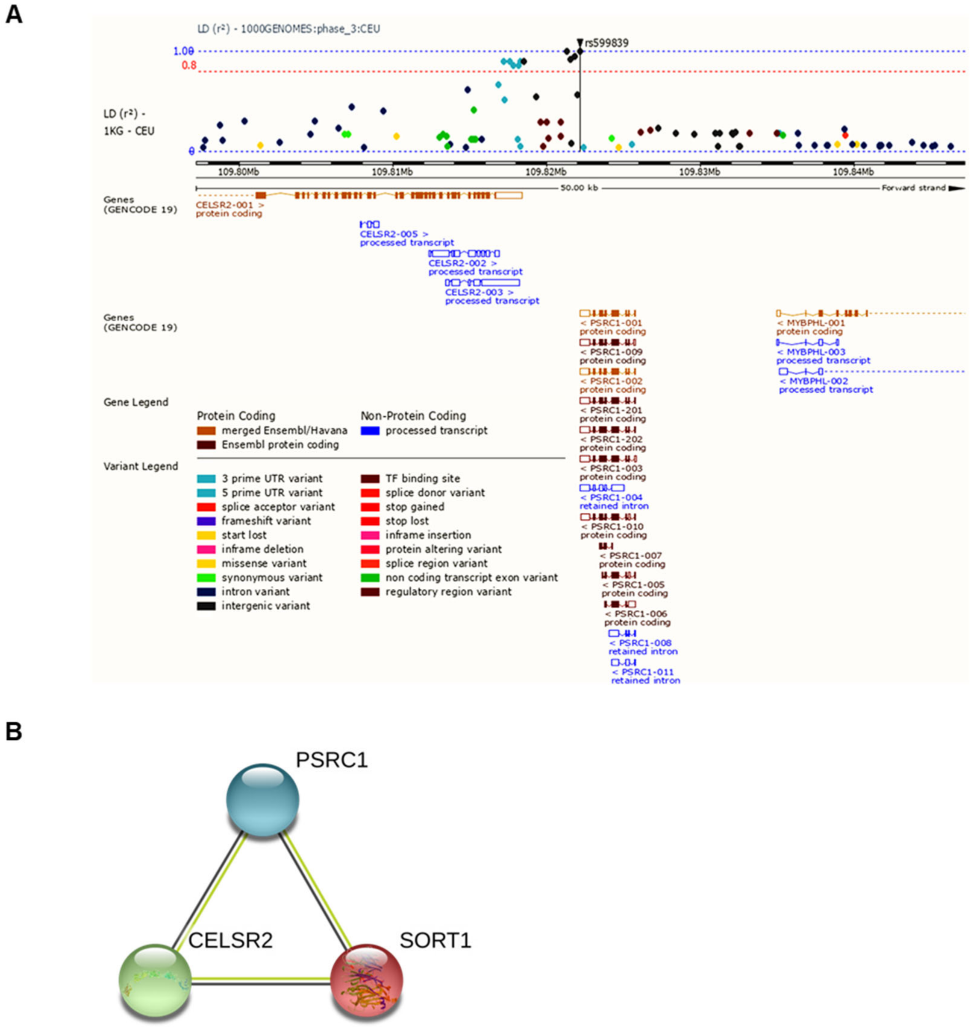

Figure S2. Genetic architecture of the region of 50.00 Kb in the 1p13.3 locus and LD of the rs599839 variant. LD analysis of the rs599839 variant and common SNPs (MAF≥0.01) located in the 1p13.3 locus, at the region spanning 50.00 Kb (chr1: 109,800,000-109,850,000; Human (GRCh37.p13)), using data from 1000 genomes project, that includes 503 individuals of European descent (CEU). Manhattan plot represents the SNPs located in the region spanning from 3′UTR of *CELSR2,* the intergenic region and 3′UTR of *PSRC1* oriented in opposite direction (on x axis). All SNPs are plotted considering r^2 values (r^2, on y axis). Dashed red line represents the cutoff of $r^2 = 0.8$, dashed blue line represents the maximum $r^2 = 1$. SNPs with $r^2 > 0.8$ are considered in LD (**A**). Network analysis with PSRC1 protein was conducted by using STRING, a web-based bioinformatic tool that show protein-protein (nodes) interactions (edges). Filled nodes represent proteins with known 3D structure. Green lines: text-mining; Black lines: co-expression. Empty node represents protein with unknown 3D structure. Protein-protein interaction enrichment was automatically calculated. $p < 0.0001$ (**A**). Connection CELSR2 – PSRC1 score of estimated probability of interaction: 0.876; Connection CELSR2 – SORT1 score: 0.778; Connection PSRC1 – SORT1 score 0.798. The network constructed indicates that these proteins has significantly more interactions than expected. Protein-protein interaction enrichment: $p = 8.44 \times 10^{-07}$. Thus, PSRC1, SORT1 and CELSR2 proteins are co-express (**B**).

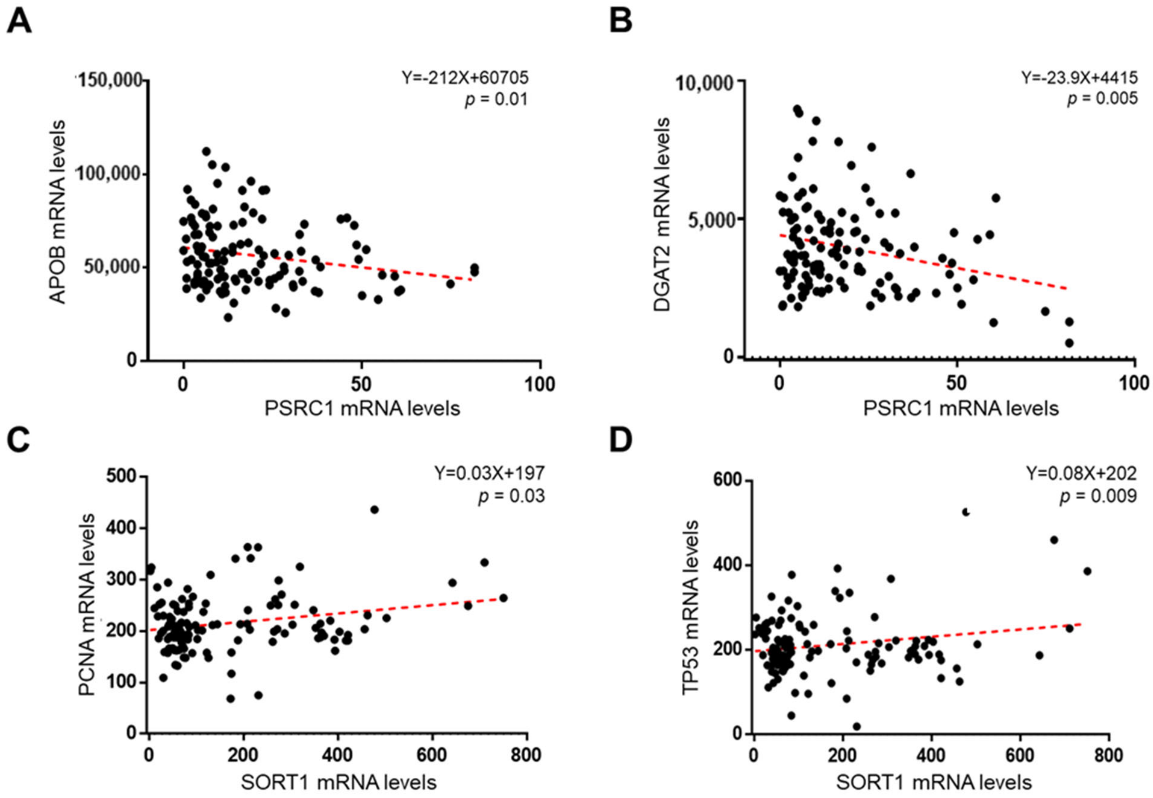

Figure S3. PSRC1 and SORT1 correlation analyses. Correlation analyses between hepatic PSRC1 gene expression and APOB (**A**) and DGAT2 (**B**) mRNA levels in liver biopsies of patients belonging to the overall cohort and evaluated by transcriptome analysis (n = 125). Correlation analyses between hepatic SORT1 gene expression and PCNA (**C**) and TP53 (**D**) mRNA levels in liver biopsies and evaluated by transcriptome analysis (n = 125).

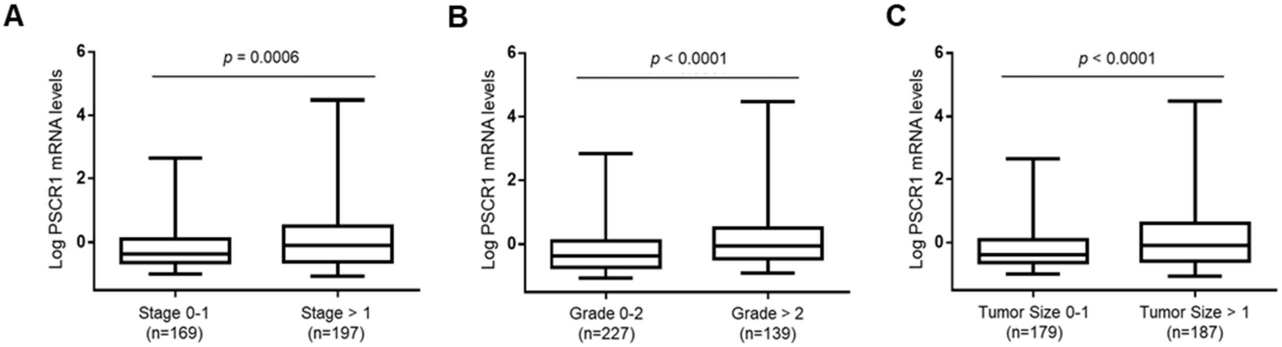

Figure S4. PSRC1 mRNA levels increases with tumor stage, grade and size. PSRC1 mRNA levels were evaluated by transcriptome analysis on HCC samples from TCGA dataset (n = 366) and stratified by advanced tumor stage (**A**), grade (**B**) and size (**C**). mRNA levels were represented as log transformed. Boxes span from 25° to 75° percentile, while whiskers indicate the 10° and 90° percentile. $P < 0.0001$ at two-tailed Student T-test.

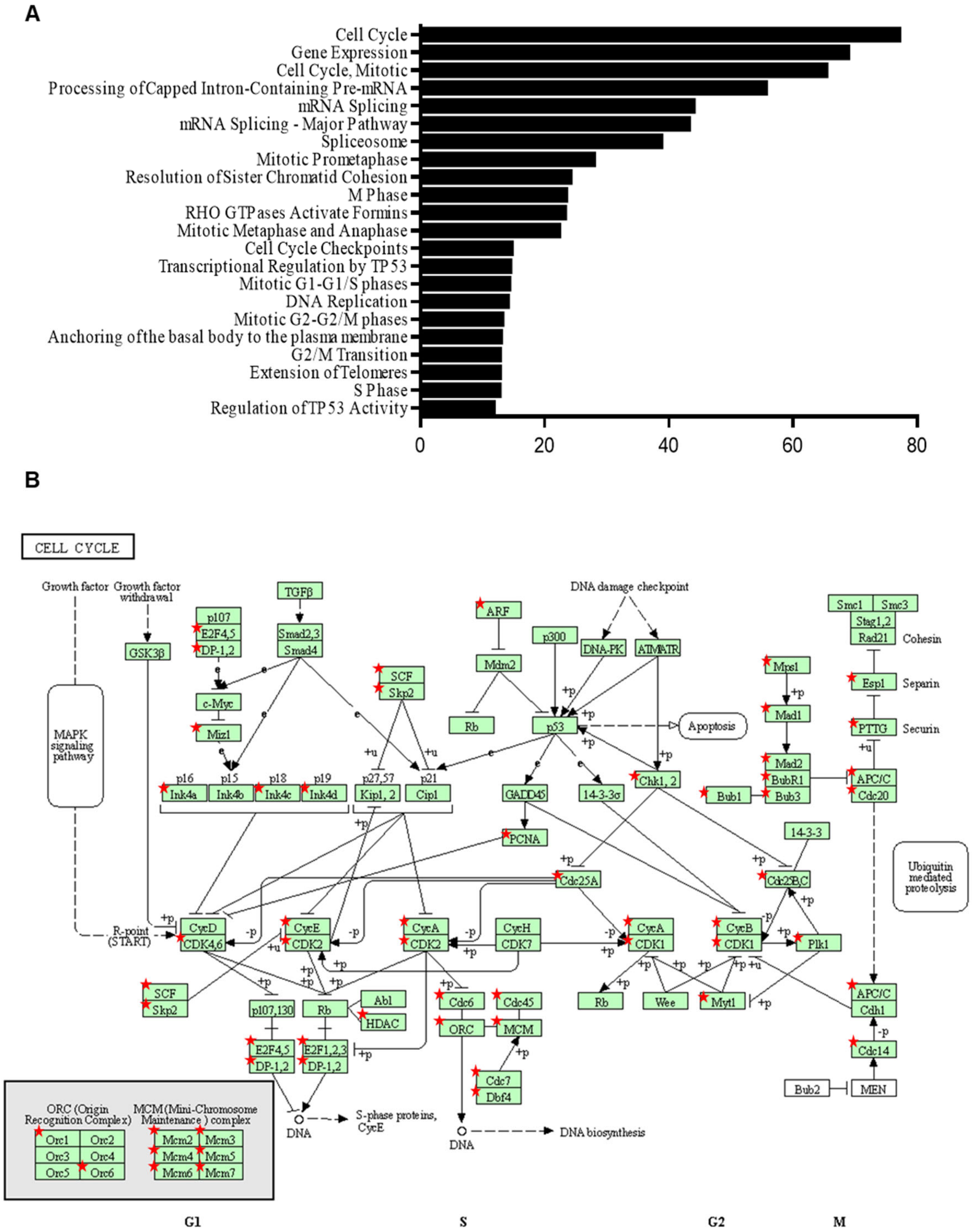

Figure S5. PSRC1 overexpressing patients up-regulate pathways mainly involved in cell proliferation and cell cycle progression. Reactome pathways enriched for 2129 genes up-regulated in patients with PSRC1 altered expression among TCGA dataset. The statistical significance level (*p*-Value) was negative 10-based log trans-formed (**A**). Schematic representation of cell cycle generated by KEGG. Red stars highlight genes up-regulated in patients with PSRC1 altered expression (**B**).

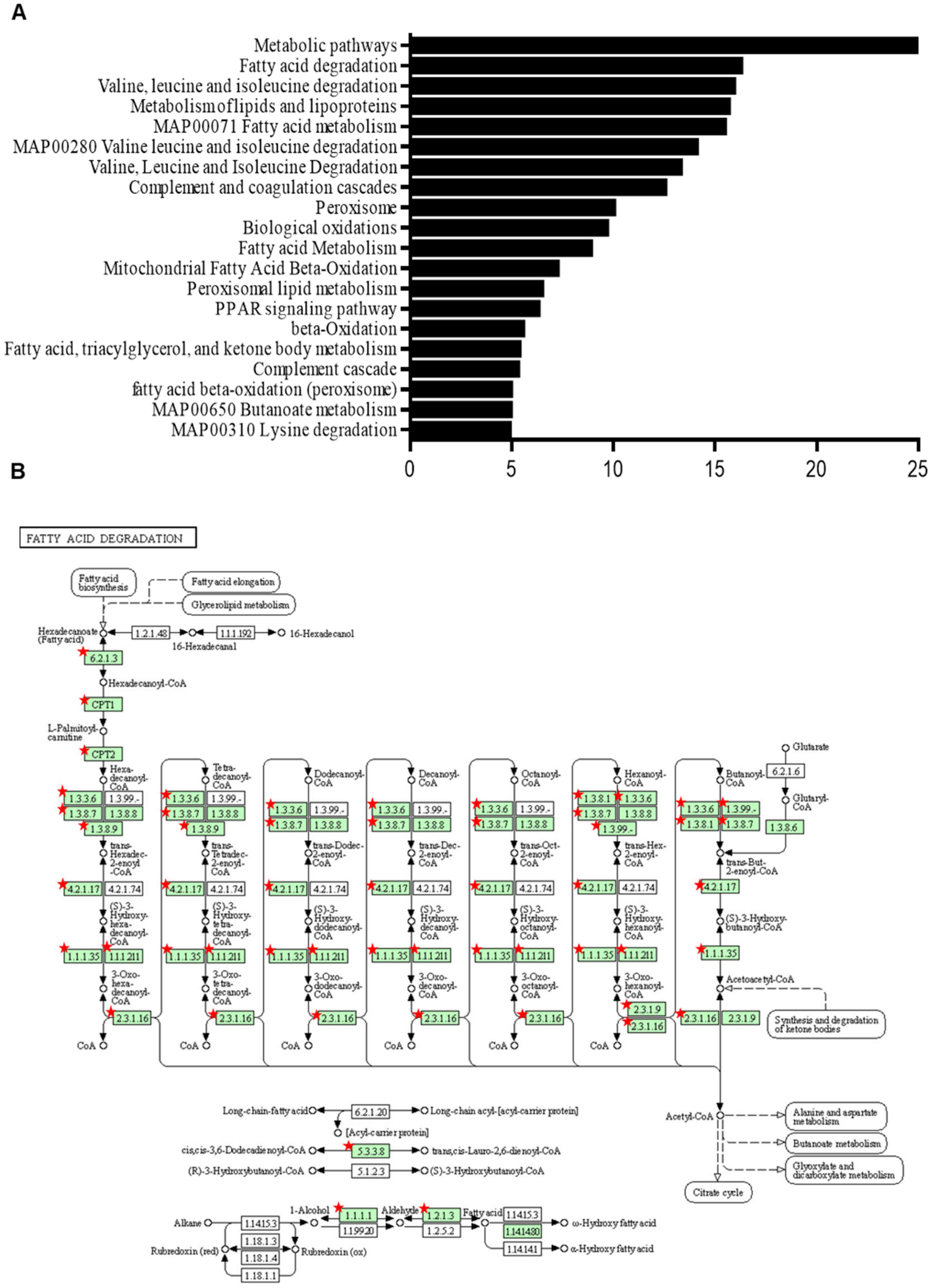

Figure S6. PSRC1 overexpressing patients down-regulate pathways mainly implicated in metabolic processes and fatty acid oxidation. Reactome pathways enriched for 1665 genes down-regulated in patients with PSRC1 altered expression among TCGA dataset. The statistical significance level (*p*-Value) was negative 10-based log trans-formed (**A**). Schematic representation of fatty acid oxidation process generated by KEGG. Red stars highlight genes down-regulated in patients with PSRC1 altered expression (**B**).
